# Cryo-EM structure of the complete and ligand-saturated insulin receptor ectodomain

**DOI:** 10.1083/jcb.201907210

**Published:** 2019-11-14

**Authors:** Theresia Gutmann, Ingmar B. Schäfer, Chetan Poojari, Beate Brankatschk, Ilpo Vattulainen, Mike Strauss, Ünal Coskun

**Affiliations:** 1 Paul Langerhans Institute Dresden of the Helmholtz Zentrum Munich at the University Hospital and Faculty of Medicine Carl Gustav Carus of Technische Universität Dresden, Dresden, Germany; 2 German Center for Diabetes Research, Neuherberg, Germany; 3 Department of Structural Cell Biology, Max Planck Institute of Biochemistry, Munich, Germany; 4 Department of Physics, University of Helsinki, Helsinki, Finland; 5 Computational Physics Laboratory, Tampere University, Tampere, Finland; 6 Department of Anatomy & Cell Biology, McGill University, Montreal, Quebec, Canada

## Abstract

Glucose homeostasis and growth essentially depend on the hormone insulin engaging its receptor. Despite biochemical and structural advances, a fundamental contradiction has persisted in the current understanding of insulin ligand–receptor interactions. While biochemistry predicts two distinct insulin binding sites, 1 and 2, recent structural analyses have resolved only site 1. Using a combined approach of cryo-EM and atomistic molecular dynamics simulation, we present the structure of the entire dimeric insulin receptor ectodomain saturated with four insulin molecules. Complementing the previously described insulin–site 1 interaction, we present the first view of insulin bound to the discrete insulin receptor site 2. Insulin binding stabilizes the receptor ectodomain in a T-shaped conformation wherein the membrane-proximal domains converge and contact each other. These findings expand the current models of insulin binding to its receptor and of its regulation. In summary, we provide the structural basis for a comprehensive description of ligand–receptor interactions that ultimately will inform new approaches to structure-based drug design.

## Abstract (precis)

The cryo-EM structure of the complete insulin receptor ectodomain saturated with four insulin ligands reveals a T-like conformation with converging membrane-proximal domains and structural evidence of insulin binding sites 2/2′.

## Graphical Abstract

**Figure:**
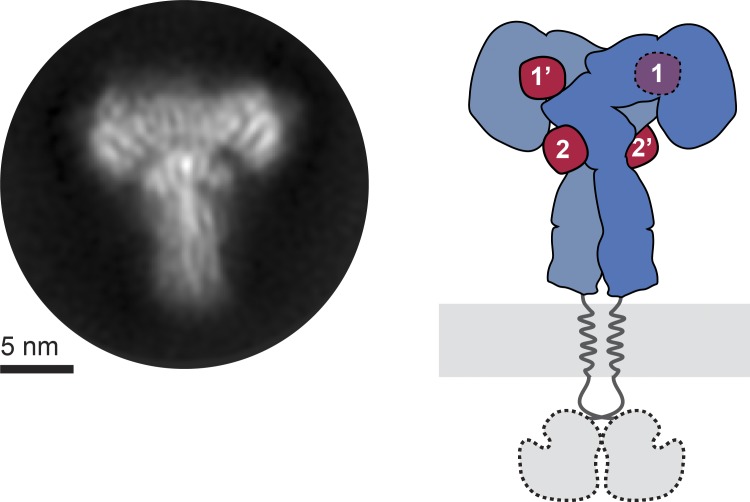

## Introduction

The insulin receptor (IR) signaling system is a key regulator of metabolism and cellular growth. Its dysfunction is linked to clinical manifestations such as diabetes mellitus, cancer, and Alzheimer’s disease (Saltiel and Kahn, 2001; Belfiore and Malaguarnera, 2011; Kleinridders et al., 2014). The IR is an extensively glycosylated disulfide-linked (αβ)_2_ homodimer with a modular domain structure. Each protomer consists of an extracellular ligand-binding α subunit and the membrane-spanning β subunit, which also harbors the intracellular kinase domain. The modular organization of the ectodomain (ECD) with high intrinsic flexibility poses a challenge to structural studies of the IR, as do the branched sugars of the glycosylation sites, and its complex ligand binding properties. Insulin binding to the ECD concomitantly elevates the receptor’s intrinsic tyrosine kinase activity before cellular signal transduction (Kasuga et al., 1982). The precise mechanism of how insulin initially engages its receptor, as well as the associated conformational changes leading to tyrosine kinase signaling, still remain elusive (De Meyts, 2015; Tatulian, 2015).

Crystallography of the unliganded (i.e., *apo*) IR-ECD dimer has revealed a structure resembling an inverted U or V with respect to the membrane, placing the membrane insertion sites ∼115 Å apart from each other (McKern et al., 2006; Croll et al., 2016). Single-particle EM of full-length IR in lipid nanodiscs corroborated that this *apo*-conformation is retained in the membrane context (Gutmann et al., 2018). Insulin binding converts the receptor ECD into a T-like shape that draws the membrane-proximal fibronectin domains closer together, enabling transmembrane signaling (Gutmann et al., 2018). Due to the low resolution of the negative-stain 2D class averages, no structural information about the location and number of bound insulin molecules could be obtained. The T-shaped conformation was confirmed shortly after by cryo-EM of the IR-ECD in complex with one or two insulins bound to the N-terminal domains (Scapin et al., 2018). However, major parts of the fibronectin domains could not be reconstructed, preventing conclusions on the transmembrane signaling mechanism.

In another cryo-EM approach, the soluble ECD was fused to a C-terminal leucine zipper (termed IRΔβ-zip) in an attempt to reduce conformational heterogeneity and to mimic membrane anchorage, thus restoring insulin-binding properties of the complete receptor (Hoyne et al., 2000; Weis et al., 2018). Structural heterogeneity was further decreased by deglycosylation and complexation with Fv variable domain modules of the anti-IR antibody 83-7. These modifications enabled the capture of a singly liganded transition state with insulin bound to the N-terminal region and with the fibronectin regions in a pincer-like fashion (Weis et al., 2018).

Previous biochemical and mutagenesis experiments have mapped two distinct binding sites, termed sites 1 and 2, on both the IR and on insulin (De Meyts et al., 1978; De Meyts, 2015). While site 1 ligand–receptor interactions were largely confirmed (Menting et al., 2013, 2014; Scapin et al., 2018; Weis et al., 2018), the structural basis of site 2 interactions remained controversial.

Here, by applying single-particle EM and atomistic molecular dynamics (MD) simulations, we report the structure of the complete, pseudosymmetric human IR-ECD in a T-like conformation saturated by four insulins. We observe that the membrane-proximal fibronectin domains converge, highlighting the coupling of ligand binding and fibronectin domain interactions as intrinsic features of the IR-ECD. While two of the observed insulin binding sites agree with those mapped in the “head” region (Scapin et al., 2018; Weis et al., 2018), the additional two insulin molecules are located in the now fully resolved “stalk” regions, providing unambiguous structural evidence for the existence and mechanism of site 2 binding.

## Results

### Purification and biochemical characterization of the complete human IR-ECD

**Figure 1.**
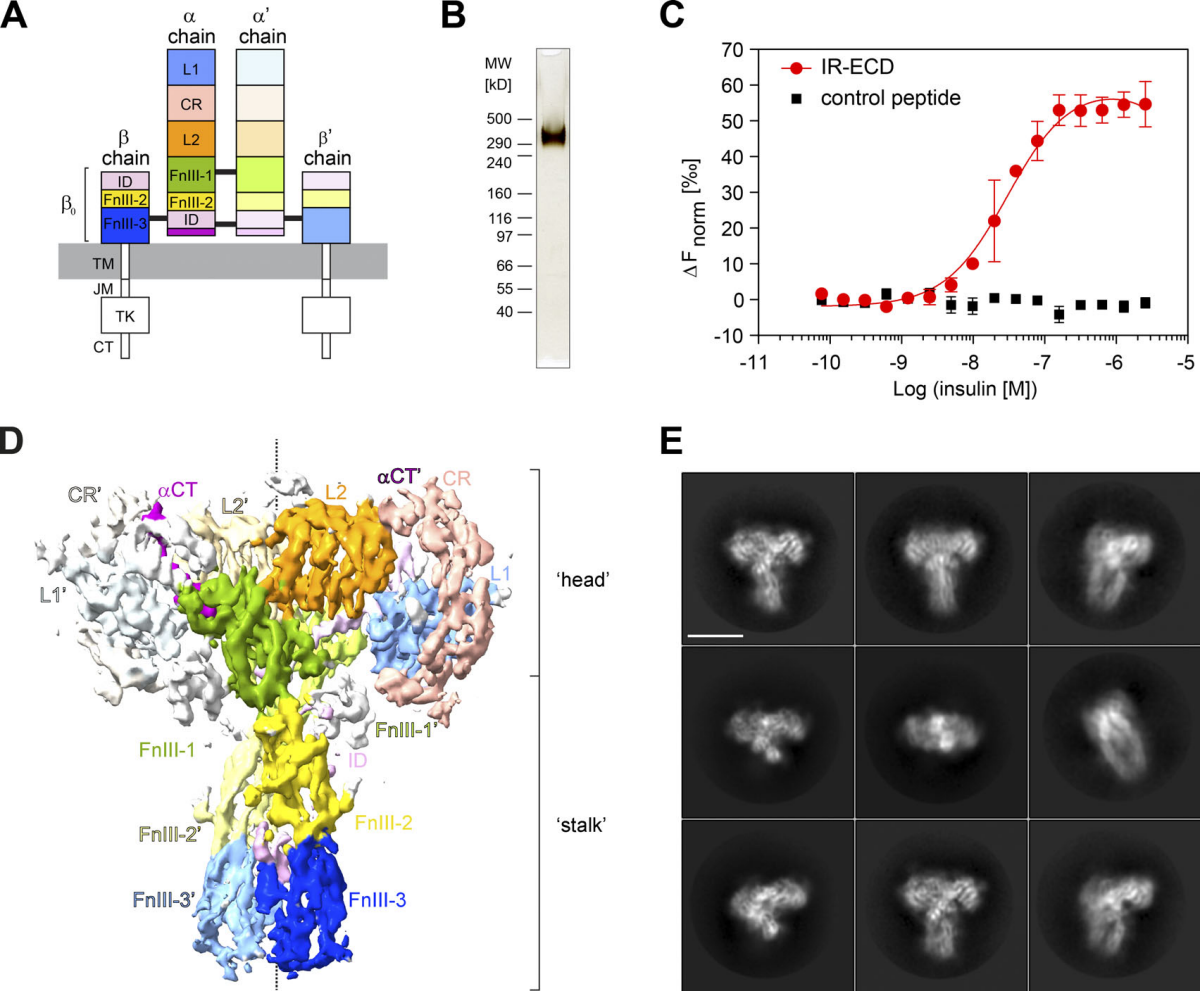
**IR-ECD purification and cryo-EM.** **(A)** Scheme of IR domain architecture. L1 and L2, leucine-rich repeat domains 1 and 2; CR, cysteine-rich domain; FnIII-1, -2, -3, fibronectin type-III domains 1, 2, 3; TM, transmembrane; JM, juxtamembrane; TK, tyrosine kinase domain; CT, C-terminal tail. The α C-terminal regions (αCT and αCT′) are drawn in purple. Black lines indicate intersubunit disulfide bonds. A prime (′) denotes the chain, domain, or residue within the second protomer. **(B)** Purified dimeric IR-ECD (IR(αβ_0_)_2_) migrates as a single band with an apparent molecular weight of 351 kD on a nonreducing 3–8% Tris-acetate SDS-PAGE gel as visualized by silver staining. **(C)** Equilibrium binding to native human insulin in solution was assessed by MST of IR-ECD after Tris-NTA-RED labeling. An 8xHis-tagged control peptide served as negative control to rule out unspecific binding or interference with the Tris-NTA-RED dye (Fig. S1 H). The normalized fluorescence difference (ΔF_norm_) is plotted against ligand concentration. Error bars display standard deviations; *n* = 3. **(D)** Front view of the IR-ECD cryo-EM density map saturated with insulin ligands at 4.3 Å estimated nominal global resolution. Subdomains are colored as in A. **(E)** Representative 2D class averages of particles contributing to the reconstruction in D of the IR-ECD exposed to human insulin. Scale bar, 10 nm.

**Figure S1.**
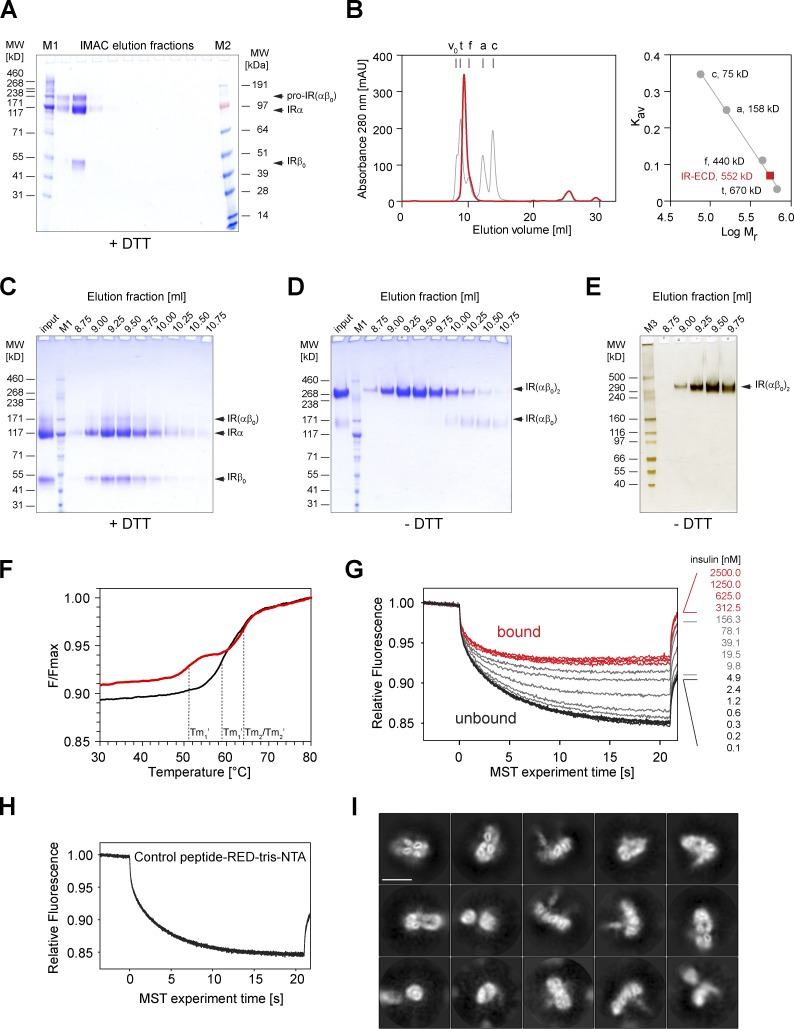
**Purification and biochemical characterization of IR-ECD.** **(A)** Coomassie G-250 Brilliant Blue–stained 4–12% Bis-Tris gel run in MOPS buffer of the IMAC elution fractions under reducing conditions. **(B)** The peak fraction containing IR-ECD was further purified by size exclusion chromatography on a Superdex 200 Increase 10/300 GL column. The void volume (v_0_) and elution volumes of the standards bovine thyroid thyroglobulin (t), horse spleen ferritin (f), rabbit muscle aldolase (a), and egg white conalbumin (c) are indicated. The partition coefficient (*K_av_*) is plotted against the logarithm of molecular weight for standards (right) to determine the IR-ECD apparent molecular weight, which is considerably larger than in denaturing SDS-PAGE, presumably due to its elongated shape in solution. **(C and D)** Samples of eluted fractions were analyzed by SDS-PAGE on 3–8% Tris-Acetate gels under reducing (C) and nonreducing (D) conditions, stained with Coomassie G-250 BrilliantBlue. **(E)** Silver-stained 3–8% Tris-Acetate SDS-PAGE gel corresponding to the single lane shown in Fig. 1 B. The apparent molecular weight was estimated to be 351 kD for IR-ECD (IR(αβ_0_)_2_), 120–130 kD for the α subunit (IRα), and 50–54 kD for the extracellular IR β (IRβ_0_) subunit as estimated with HiMark unstained protein standards (M3). Other markers used here were HiMark prestained protein standard (M1) and SeeBlue Plus2 prestained protein standard (M2). **(F)** Thermal unfolding of IR-ECD was assessed in the absence (black) or presence (red) of 50 µM human insulin by recording intrinsic tryptophan autofluorescence ratios at 350 and 330 nm. The plot shows temperature-dependent normalized tryptophan autofluorescence. The melting temperatures in the absence of insulin (T_m1_, T_m2_) and in the presence of insulin (T_m1_′, T_m2_′) are indicated. **(G)** Representative MST traces of 10 nM IR-ECD (labeled with RED-Tris-NTA) after exposure to insulin. Native insulin at concentrations from 2.5 µM to 76 pM was titrated against 10 nM soluble RED-Tris-NTA–labeled IR-ECD. The corresponding dose–response curve is plotted in Fig. 1 C. **(H)** To rule out nonspecific interactions or interference with the labeling strategy, MST traces of a synthetic control peptide labeled with RED-Tris-NTA were recorded after exposure to insulin (same concentrations as in G) and confirmed not to interact with insulin at concentrations ≤2.5 µM. **(I)** 2D class averages of the *apo*-IR-ECD obtained by cryo-EM. Scale bar, 10 nm.

The complete IR-ECD (IR(αβ_0_)_2_; Fig. 1 A) was produced by secretion from human embryonic kidney cell–derived cells, ensuring human-like posttranslational processing, such as glycosylation. Purification of the recombinant protein directly from the medium resulted in a highly pure IR-ECD that was amenable to cryo-EM studies. SDS-PAGE confirmed a complete, glycosylated, dimeric polypeptide composition with an apparent molecular weight of 351 kD (Figs. 1 B and S1). Ligand binding was assessed by two independent assays in solution without diffusion constraints (Figs. 1 C and S1 F), and thus comparable to our cryo-EM experiments. In both of these assays, ligand labeling was entirely omitted to preserve binding properties. First, the thermal stability of purified IR-ECD was followed by low-volume differential intrinsic tryptophan scanning fluorimetry with a temperature gradient from 20°C to 95°C (Fig. S1 F). In the absence of insulin, IR-ECD unfolds in two steps, with transition temperatures of 58.9°C and 64.0°C. Interestingly, insulin binding shifted the first transition temperature down to 51.1°C, implying that insulin binding leads to conformational changes within distinct regions of the IR-ECD. Next, ligand binding affinity measured by microscale thermophoresis (MST; Seidel et al., 2013) showed an equilibrium dissociation constant of *K*_d_ = 30.0 ± 4.3 nM (Figs. 1 C and S1 G), corresponding to a low-affinity binding regimen. This is in good agreement with the established concept that the soluble IR-ECD lacking membrane anchorage loses high-affinity binding in the picomolar range (Whittaker et al., 1994, 2008; Bass et al., 1996; Kiselyov et al., 2009; Subramanian et al., 2013; De Meyts, 2015), similar to the EGF receptor ECD (Lax et al., 1991; Ferguson et al., 2003).

### Single-particle cryo-EM analysis of the IR-ECD

The IR-ECD was analyzed by single-particle cryo-EM in the absence and presence of recombinant human insulin. Vitrification conditions allowed cryo-EM data collection for the unliganded as well as for the liganded ECD. In the absence of ligand, 2D class averaging revealed considerable structural heterogeneity (Fig. S1 I). Although individual domains could be identified in a subset of 2D class averages, no high-resolution features, such as clearly identifiable, individual secondary structural elements, were apparent. Consequently, attempts at reconstructing these data in 3D did not yield any subnanometer EM maps. In particular, the fuzzy haphazard appearance of membrane-proximal fibronectin domains in the 2D class averages points at considerable flexibility. This behavior of the IR-ECD in isolation most likely reflects the presence of various transition states and conformations sampled in the absence of insulin.

For cryo-EM samples of the liganded IR-ECD, saturating amounts of 40 µM insulin were used, corresponding to a dimeric receptor IR(αβ_0_)_2_:ligand molar ratio of ∼1:28. The rationale for such a large ligand excess was to ensure saturation of all available insulin binding sites on the receptor and the associated reduction of structural heterogeneity. The insulin concentration used here is in a similar micromolar range as in previous cryo-EM studies owing to the required IR-ECD protein concentration for the cryo-EM analysis. Scapin et al. (2018) incubated IR-ECD with 28 µM insulin before the EM analysis, while Weis et al. (2018) eluted IRΔβ-zip from an insulin-affinity column with 50 µM insulin before separating the insulin-bound complex from free insulin by gel filtration. In summary, insulin concentrations used throughout these studies are high compared with physiological insulin concentrations of up to ∼5 nM, depending on location and metabolic state (Horwitz et al., 1975).

**Figure S2.**
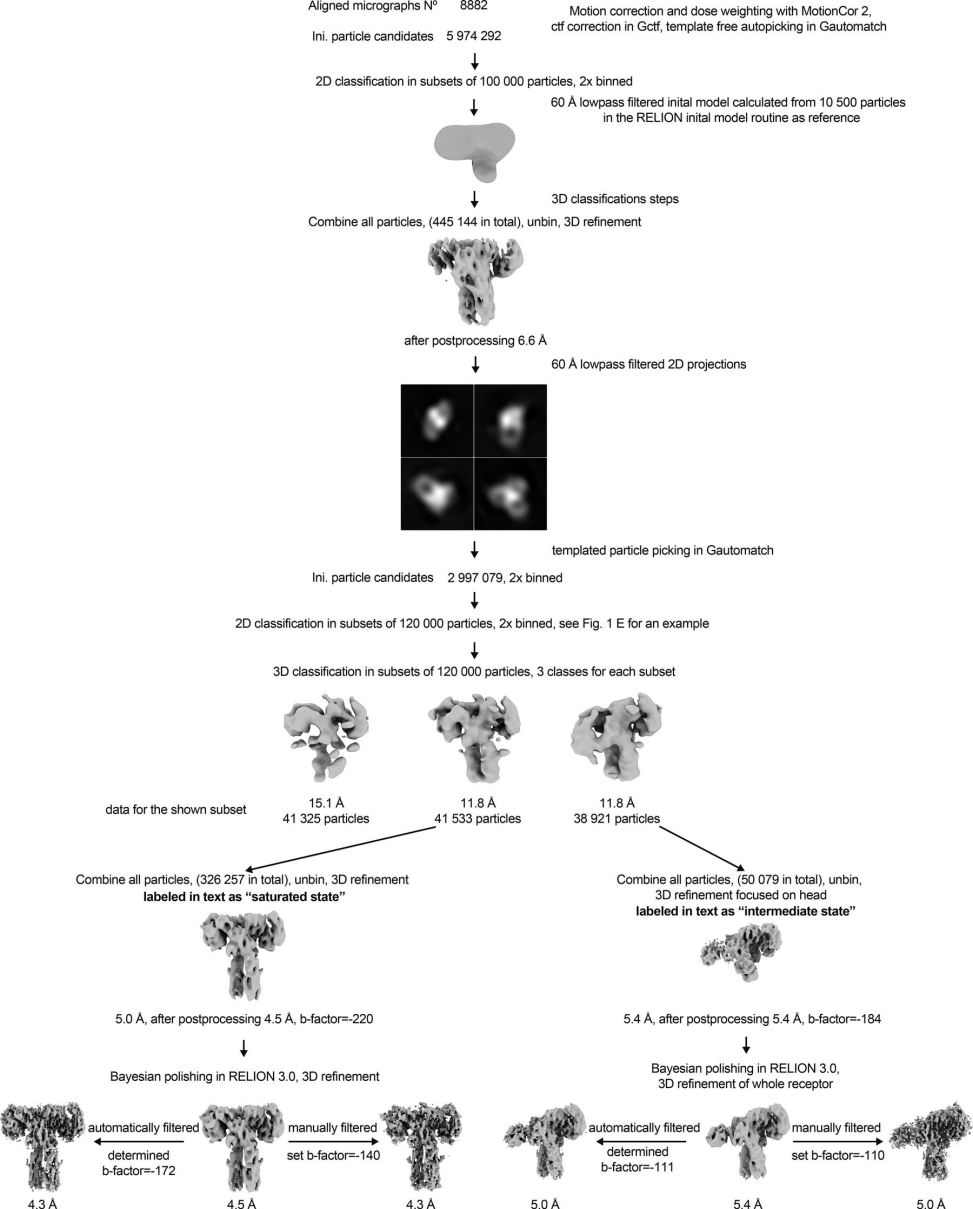
**Overview of the cryo-EM data processing scheme.** Particle sorting and classification scheme used for 3D reconstruction of the insulin–IR-ECD complex. The individual nominal global resolutions are quoted as good proxies for translational and rotational accuracy of reconstructions as well as for the level of detail observed in individual maps. Ini, initial.

**Figure S3.**
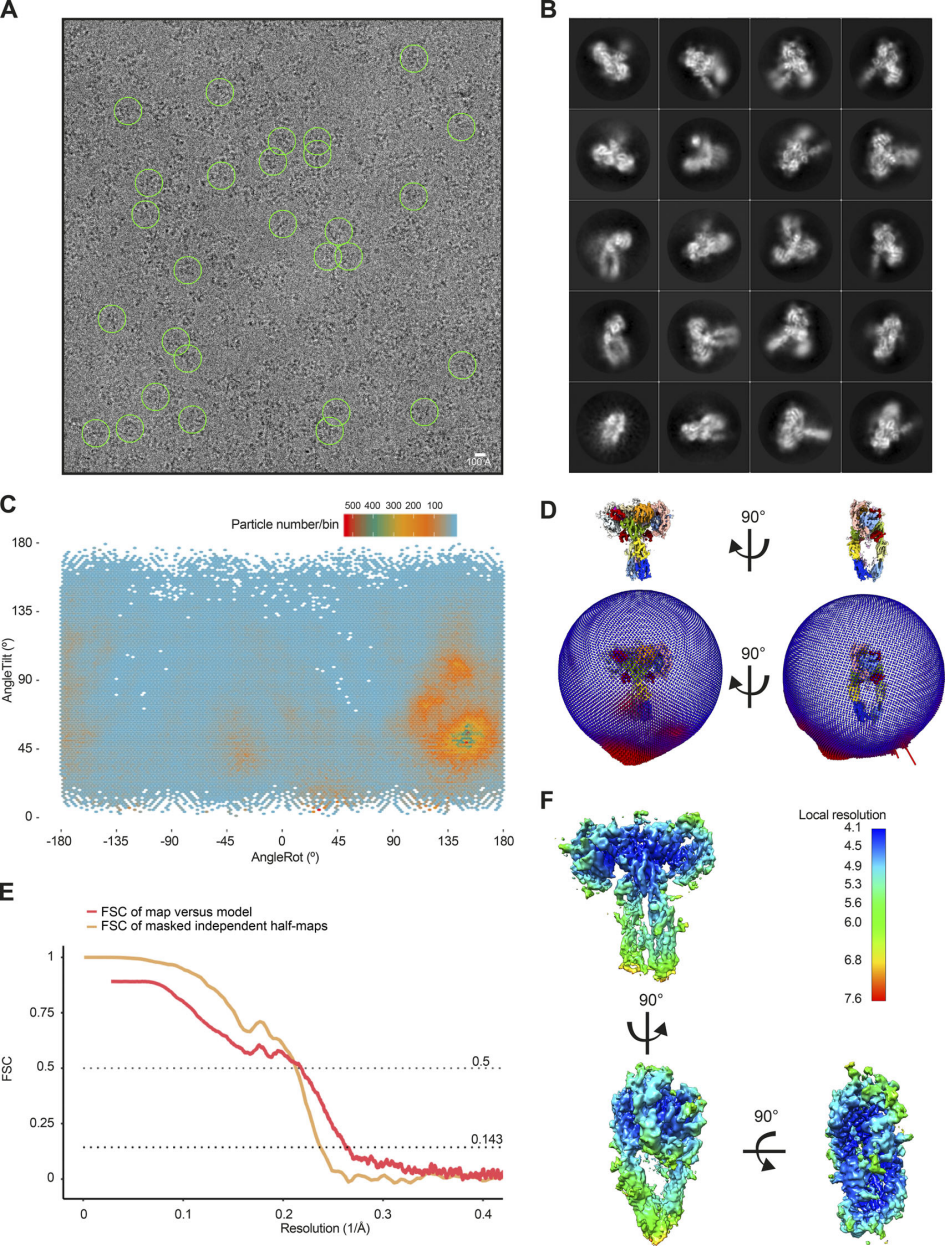
**Single-particle cryo-EM analysis of the insulin–IR-ECD complex.** **(A)** Representative micrographs of the insulin–IR-ECD dataset. The scale bar in the cryo-EM micrograph corresponds to 100 Å, and the green circles (260-Å diameter) indicate particles contributing to the final reconstruction with a nominal global resolution of 4.3 Å (see Fig. S2). **(B)** Reference-free 2D class averages of the insulin–IR-ECD complex from an initial 2D classification run (see Fig. S2 for details). Some structural heterogeneity is apparent, especially in the stalk region. **(C)** Angular distribution of particles contributing to insulin–IR-ECD complex reconstruction. Tilt and rotation angles were plotted against each other for the final 4.3-Å 3D reconstruction. The color of each sampling bin indicates the number of particles in the respective bin. **(D)** In the spherical angular distribution representation, blue denotes fewer, and red more, particles (326,257 particles in total). **(E)** FSC of masked independent half-maps and of map-versus-model of the final insulin–IR-ECD reconstructions used for modeling and structure interpretation (see Fig. S2 for details). The nominal global resolution of the full insulin–IR-ECD complex was determined to be 4.3 Å according to the 0.143 cutoff criterion (Rosenthal and Henderson, 2003). Map-to-model correlation showed agreement at the 0.5 cutoff criterion to 4.6 Å. **(F)** Map of the insulin–IR-ECD complex colored according to local resolution estimate. The central parts of the head are resolved at higher resolution, whereas distal parts of the stalks are resolved at lower resolution.

For the ligand-bound IR-ECD, individual secondary structure elements became clearly discernable after 2D classification (Figs. 1, S2, and S3). After further classification steps, 3D refinement, and map sharpening, the 3D reconstruction of the ligand-saturated IR-ECD reached an apparent overall resolution of 4.3 Å, as estimated by the Fourier shell correlation (FSC) of independently refined half-maps (0.143 criterion; Rosenthal and Henderson, 2003; Figs. S2 and S3). Our 3D reconstruction confirmed the T-like conformation as seen in the insulin-bound full-length IR at low resolution by negative stain EM (Gutmann et al., 2018). Features of the compact head containing L1, CR, and L2 domains appear better defined than the fibronectin stalks, which exhibit more flexibility (compare local resolution estimate in Fig. S3 F). Importantly, we have refrained from applying C2 symmetry during any of the processing steps. This strategy proved the most appropriate since initial classification in 2D and 3D indicated flexibility and a degree of asymmetry in the organization of the ligand-saturated IR-ECD (Figs. S2 and S3 B). The asymmetry between the two IR-ECD protomers is clearly reflected in the final reconstruction of the saturated state and manifests itself in the built model as described in detail below where appropriate.

**Figure 2.**
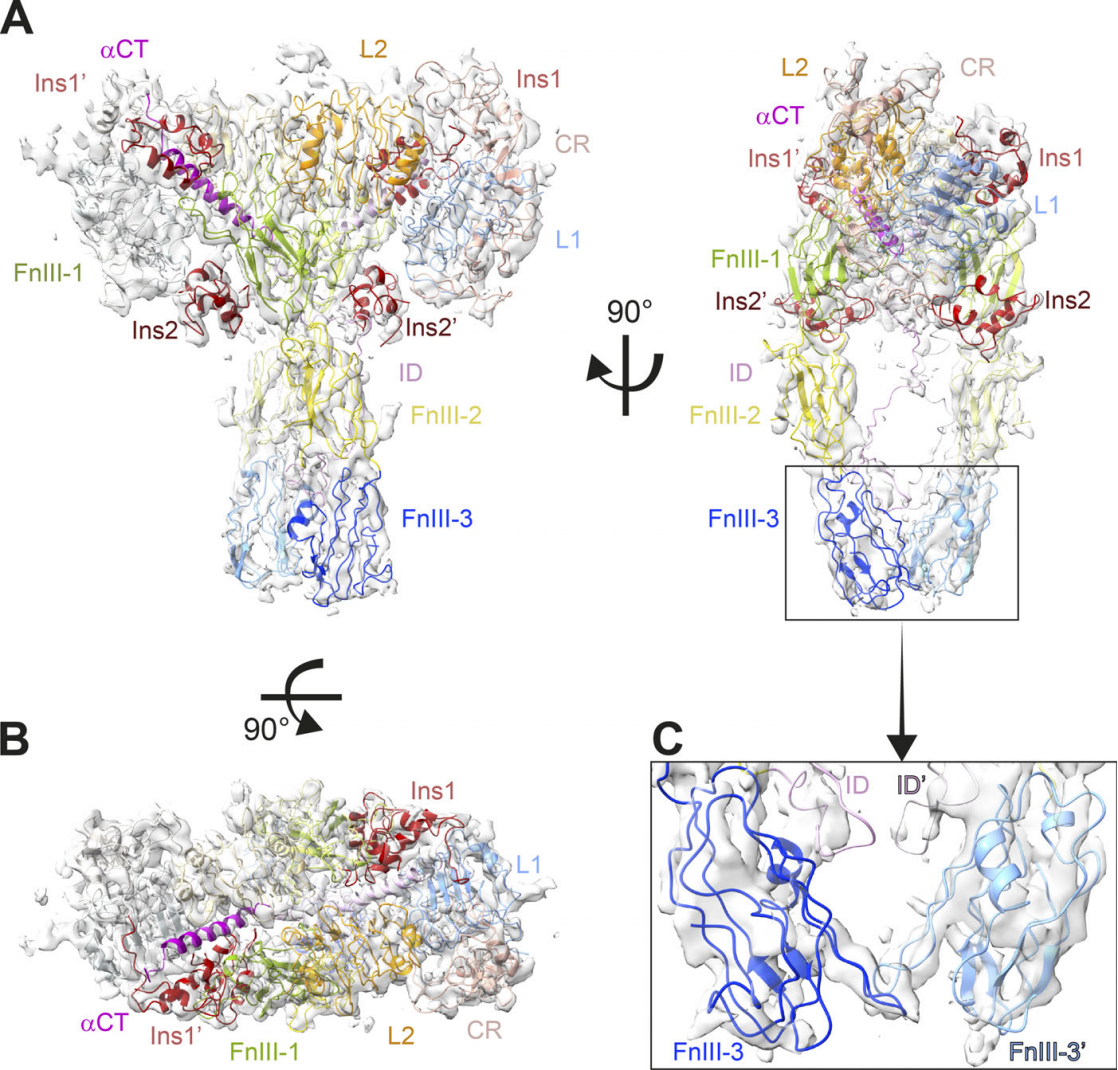
**Cryo-EM structure of the ligand-saturated IR-ECD.** **(A and B)** Orthogonal views of the cryo-EM map and structure of the IR-ECD dimer complex. **(C)** Close-up of the membrane-proximal FnIII-3 domains. The color code for the individual domains in all panels is as in Fig. 1; the four insulin moieties are depicted in red.

**Figure S4.**
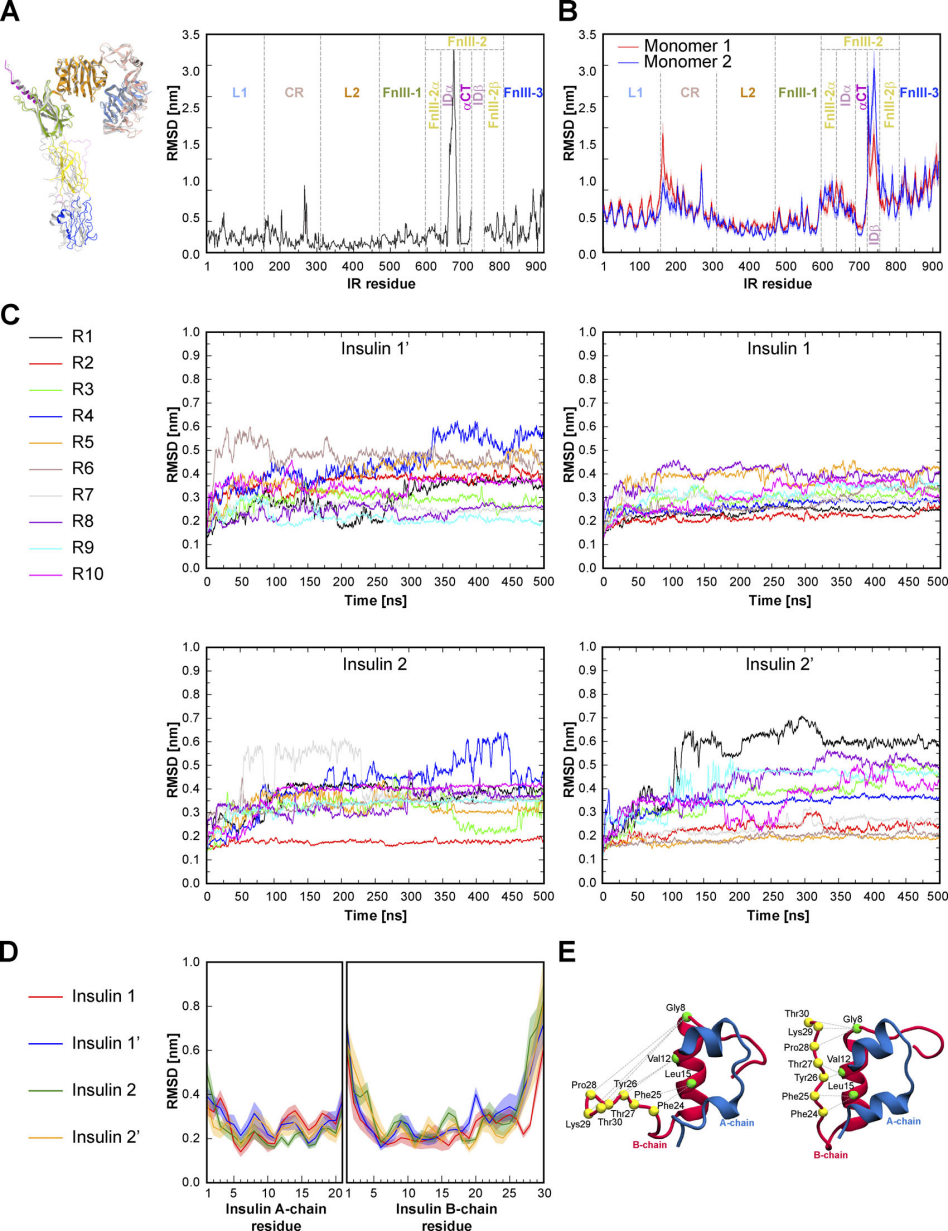
**Structural asymmetries in the cryo-EM structure and MD simulations **of the ligand-saturated IR-ECD**.** **(A)** Asymmetries depicted in our cryo-EM structure. The panel on the left shows the two superimposed IR monomers. Monomer 1 domains are colored as in Fig. 1, and monomer 2 is colored in gray. The corresponding per-residue plot of the backbone RMSD between the two monomers is shown on the right. Individual domains are separated by dashed bold lines. **(B)** Backbone RMSD measured for IR residues averaged from 10 MD simulations. Red and blue lines indicate monomer 1 and 2, respectively. RMSDs were calculated over 500 ns with respect to the starting MD model. **(C)** Time-dependent backbone RMSD for the four bound insulins determined from 10 MD simulation repeats (R1–R10). RMSDs were calculated with respect to the initial MD model. **(D)** RMSD determined for insulin residues calculated with respect to the initial MD model and averaged over 10 MD simulation runs. **(E)** To monitor the B-chain C terminus dynamics, we recorded the distance between residues in the B-chain α helix and residues within the B-chain C terminus as indicated above with dashed lines. Cryo-EM structures of insulin 1 in the open conformation (left) and insulin 2 in the closed conformation (right) are displayed in cartoon representation. See Table S4 for the corresponding distance measurements from the cryo-EM structure and from MD simulations. Standard error of the mean for B and D are indicated as shadows.

All structured domains of the IR-ECD, as well as the localization of the insulins, were unambiguously identified in our cryo-EM density map (Figs. 1 D and 2 and Tables S1 and S2). This map in combination with previously published structural information enabled us to construct a single model for IR-ECD in complex with four insulins (Fig. 2). The single exceptions were the intrinsically disordered insert domains (IDs) of the IR-ECD that could only partially be modeled into certain incohesive density features in the vicinity of the fibronectin domains. The IDs encompass the cleavage site for furin (residues 720–723, Arg-Lys-Arg-Arg), which processes the IR(αβ) polypeptide chain into the IRα and IRβ chains. Thus, the ID is separated into IDα and IDβ in the mature receptor dimer. Our data allowed us to tentatively model the IDα loop, but we refrained from including IDβ in the cryo-EM structure. To err on the side of caution, we did not model the furin cleavage sites in the cryo-EM structure, even though a noncontiguous density feature attributable to this part of the IR-ECD α′-chain is present in the map (Table S2). After initial rigid body docking and local flexible fitting of the IR-ECD domains into the EM map, the resulting structure was manually rebuilt and refined. The final model (deposited to PDB, 6SOF) conforms to commonly accepted quality indicators (Table S1). The aforementioned pseudosymmetric organization of the two IR-ECD protomers is clearly reflected in their substantial model root mean square deviation (RMSD) in certain areas (e.g., regions of the CR domain; Fig. S4 A).

### Atomistic MD simulations

**Figure 3.**
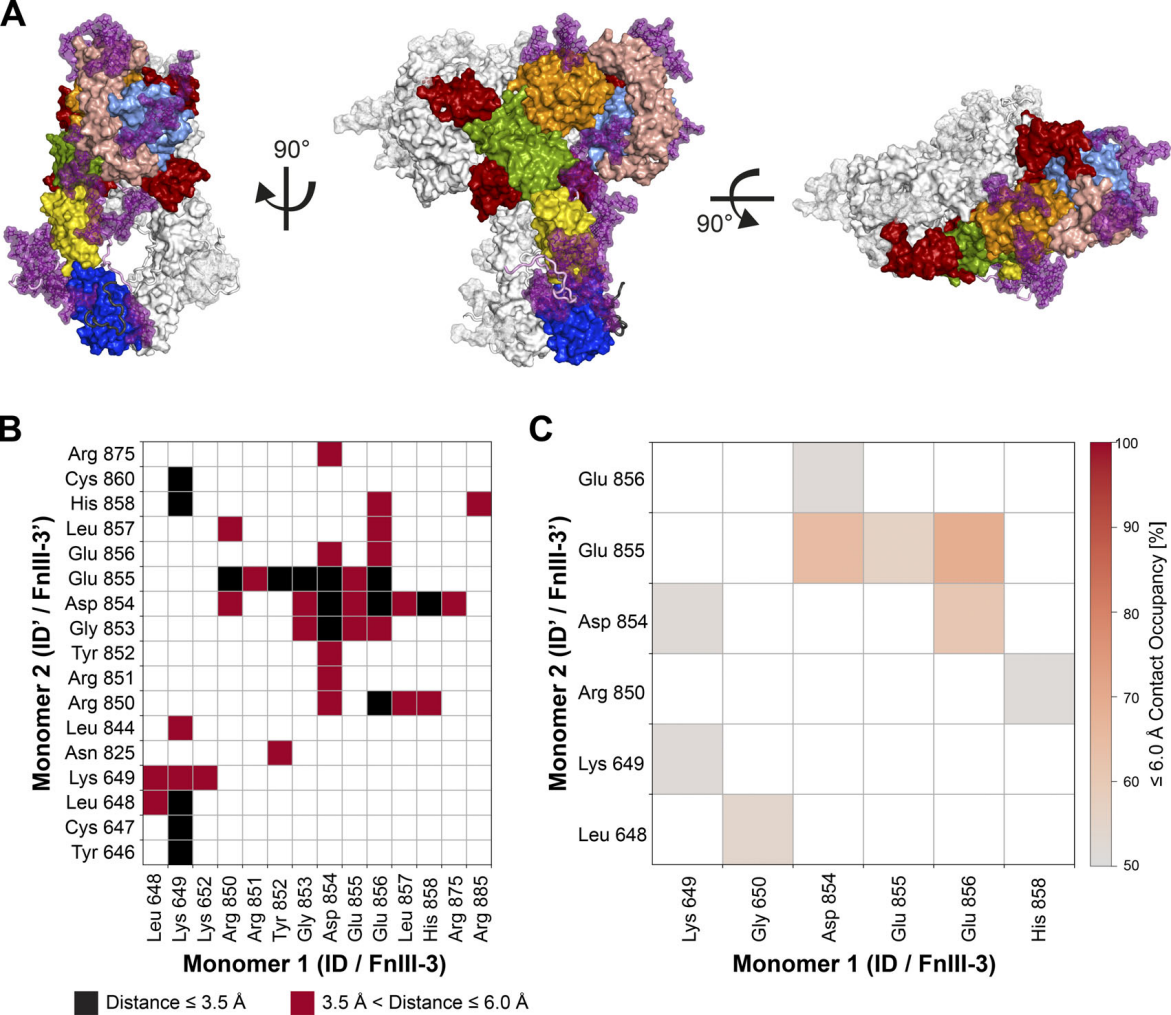
**MD simulations of insulin-saturated IR-ECD and interactions of membrane-proximal domains.** **(A)** Orthogonal views of the complete insulin–IR-ECD starting model used for MD simulations in surface representation. One monomer is color-coded as in Fig. 1 with carbohydrates in purple and the insulin moieties in red. The second monomer is depicted in white. The disordered ID and C-terminal linker plus tag are shown in cartoon style colored in light violet and dark gray, respectively. **(B)** Contact map calculated from our cryo-EM structure showing interactions between ID/FnIII-3 domains of both monomers with contact cutoffs set to ≤3.5 Å (black) and ≤6 Å (red). **(C)** Contact occupancies calculated from MD simulations with the cutoff at 6 Å. Only contacts of ≥50% occupancy are displayed.

To follow the dynamics of the insulin–IR-ECD interactions, we performed atomistic MD simulations. For the sake of completeness, we extended our experimentally determined insulin–IR-ECD model by incorporating the previously absent IDβ loops, as well as the *N*- and *O*-linked glycans based on previous reports (Sparrow et al., 2007, 2008; Fig. 3 A and Tables S2 and S3). Since furin cleaves C-terminally of this sequence and because there is no evidence of its removal in the secreted ECD, we also included the furin cleavage site to the αCT helix in our simulation models. In fact, in the case of αCT′, the furin cleavage site could be fitted into our density map; however, the resolution in this region was too low to finalize this part of the model (Table S2). This approach allowed us to calculate contacts and interactions at the atomistic level for the IR-ECD model, including all loops mentioned above and the glycans. A contact between two residues was considered to be established as a stable interaction if the distance between any pair of atoms in the two residues was ≤3.5 or ≤6 Å, and the occupancy at this distance was ≥50% of simulation time (Fig. 3 C). In 10 independent 500-ns simulations, the insulin-saturated ECD displayed increasing flexibility toward the stalks (Fig. S4 B), in line with our cryo-EM data.

### The insulin-saturated IR-ECD adopts a T-shape with converging FnIII-3 domains

In accord with previous data, significant conformational changes in the insulin-bound IR-ECD are observed with respect to rigid-body rotations in the *apo*-IR-ECD (McKern et al., 2006; Croll et al., 2016; Gutmann et al., 2018; Scapin et al., 2018). The liganded IR-ECD adopts a T-shaped conformation (Figs. 1 D and 2) similar to the membrane-embedded full-length receptor at low resolution (Gutmann et al., 2018). The fibronectin domains come together in a pincer-like fashion (Fig. 2 C) similar to what has been described for the C-terminally tethered IRΔβ-zipInsFv complex (Weis et al., 2018). The same FnIII-3 domain loops are in proximity in our structure and MD simulations as in the IRΔβ-zipInsFv structure (in particular residues Asp854–His858; Fig. 3, B and C). This contrasts with the overall completely different arrangement of the fibronectin domains in the two structures. Additionally, we observe proximity between the residues Leu648–Lys652 of ID and Tyr646′–Lys649′ of ID′ domains (Fig. 3, B and C). Thus, the membrane-proximal domains are capable of interacting in the absence of a C-terminal zipper element or membrane attachment. The interaction between the FnIII-3 and FnIII-3′ domains further supports the concept that receptor activation is directly linked to the lateral distance between transmembrane domains and consequently the relative orientation of the attached intracellular kinase domains (Kavran et al., 2014; Gutmann et al., 2018).

### Insulin binding sites 1 and 1′

Earlier biochemical studies showed that each IR protomer contains two distinct insulin-binding sites, termed site 1 and 2 (site 1′ and 2′ on the other protomer; De Meyts, 1994, 2015; Schäffer, 1994). The IR-ECD head region of our structural model agrees well with the IR-ECD cryo-EM structure in complex with two insulins in site 1/1′ (Scapin et al., 2018): the L1-CR-L2 module is complexed by one insulin and adopts an ∼90° angle with respect to the [L2-(FnIII-1):L2′-(FnIII-1′)] module, and insulin 1 interacts with the L1-αCT′ tandem element and loops of the FnIII-1′ domain (L1′+αCT and FnIII-1 in case of insulin 1′; Fig. 2). The mode of insulin binding to site 1 is essentially the same as in the previous cryo-EM structures, with details better resolved than in Scapin et al. (2018) and resolved approximately the same as in Weis et al. (2018). In addition, our MD simulations largely confirmed the previously described residue interactions of insulin 1/1′ with IR-ECD (Figs. S6 and S7, upper panels). In the simulations, however, insulin 1′ appeared to undergo more overall conformational fluctuations compared with insulin 1 (Fig. S4 C, compare RMSD plots for insulin 1′ and 1). Along this line, the C-terminal B-chain of insulin 1′ featured higher flexibility compared with insulin 1 (Fig. S4 D).

### Structural identification and characterization of insulin binding to sites 2/2′

**Figure 4.**
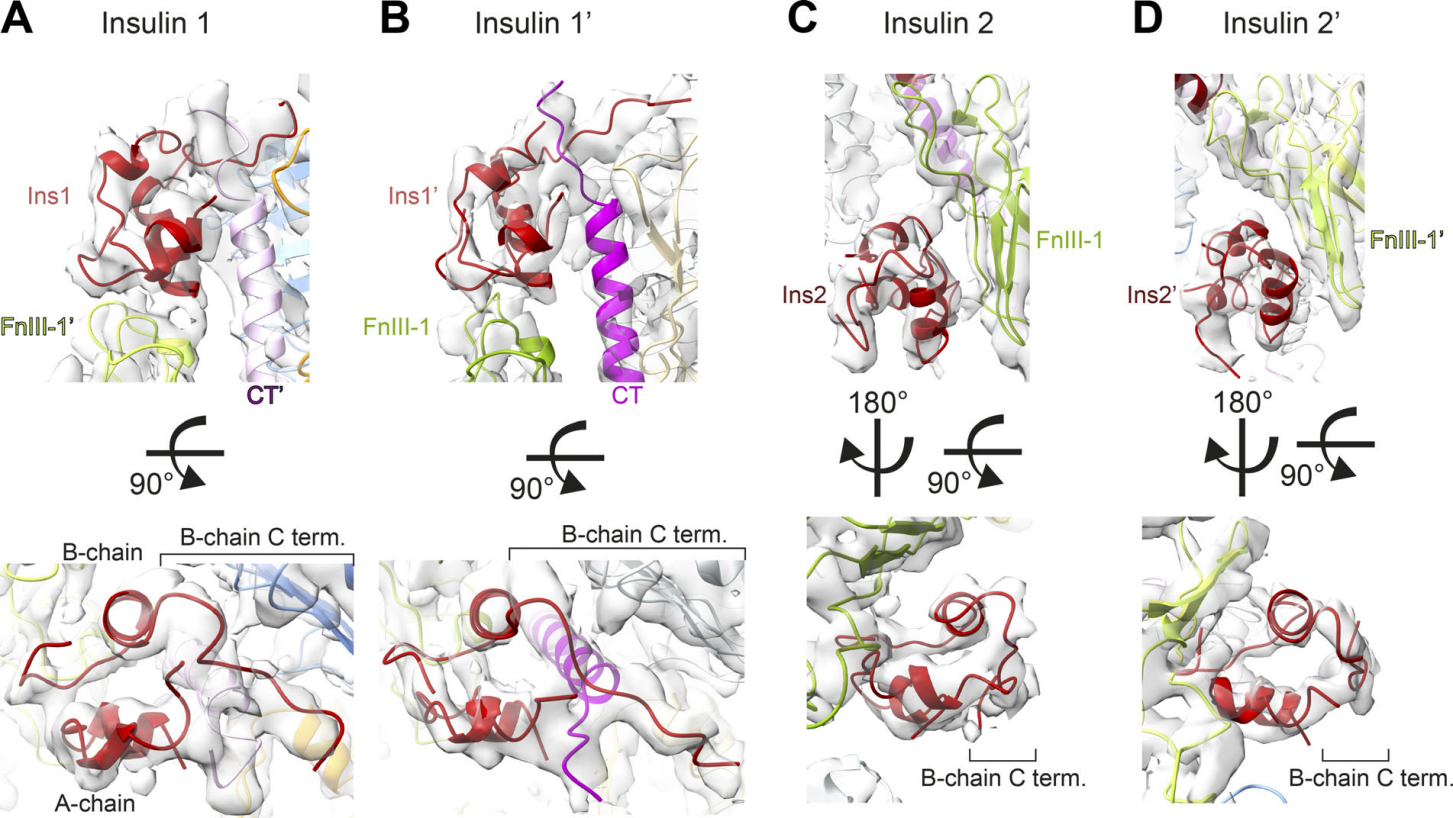
**Binding sites and conformations of insulins bound to IR-ECD.** **(A–D)** The cryo-EM density map and structure in close-up views of the four insulins and their receptor binding sites are displayed: insulin 1 (A), insulin 1′ (B), insulin 2 (C), and insulin 2′ (D). Insulin structures are aligned below for comparison illustrating the open conformation of site-1-bound insulins with a detached C-terminal B-chain segment in contrast to the closed conformation at site 2. Individual domains in all panels are colored as in Figs. 1 and 2.

**Figure S5.**
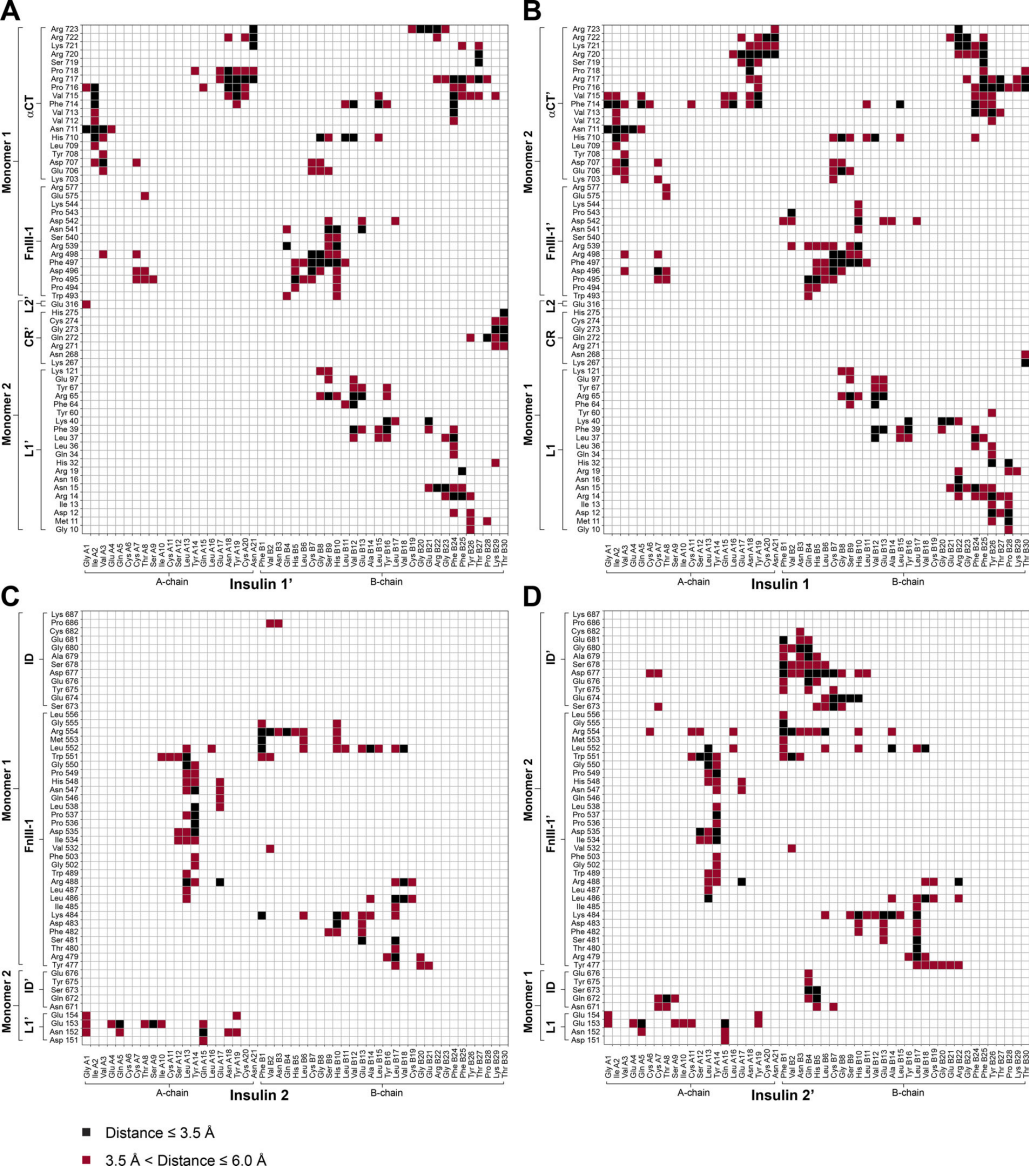
**Contact map for insulin–IR-ECD interactions in the cryo-EM structure.** **(A–D)** Contact map showing interactions between IR-ECD and head-bound insulins 1′/1 (A and B) and stalk-bound insulins 2/2′ (C and D). The arrangement of the maps corresponds to the location of the respective insulin in the ECD (front view).

Contrasting to previous structures, two additional and previously undescribed density features contacting the FnIII-1 and FnIII-1′ domains were identified in our cryo-EM map (Figs. 1 D and 2). These features did not correspond to unmodeled regions of the IR-ECD but could clearly be assigned as additional insulin molecules (Figs. 2 and 4). The concordance between these density features and insulin is further supported by the observation that the map-versus-model correlation coefficient of both head- and stalk-bound insulins is almost indistinguishable. If anything, it is slightly inferior for the head-bound ligands (Table S1). These additional insulin molecules were analogously named insulin 2 and 2′, as they employ their site 2 residues to interact with β-sheets and interstrand loops (Tyr477–Trp489 and Asp535–Arg554) of the FnIII-1 and FnIII-1′ domain, respectively (Figs. 4 and S5). Among those FnIII-1 residues, Lys484 and Leu552 have been implicated in insulin binding to site 2 in the holoreceptor by alanine scanning mutagenesis screens (Whittaker et al., 2008). Also, insulins 2 and 2′ interact with a loop within the L1′ (Asp151′–Glu154′) and L1 domain (Asp151–Glu154), respectively (Fig. S5).

Interestingly, head- and stalk-bound insulins display different conformations. Insulin consists of two polypeptide chains, an A-chain of 21 residues structured into two α-helices separated by a stretch of extended polypeptide and a B-chain of 30 residues with a central α-helix (Adams et al., 1969). Insulins 1 and 1′ were in the receptor-bound “open” conformation with a detached B-chain C terminus (including the aromatic triplet Phe B24, Phe B25, and Tyr B26) that is critical for engaging receptor site 1 (Hua et al., 1991; Menting et al., 2014). In contrast, insulins 2 and 2′ have a “closed” conformation, which corresponds to the conformation in solution before IR binding (Hua et al., 1991; Fig. 4, bottom panels; and Fig. S4 E).

**Figure 5.**
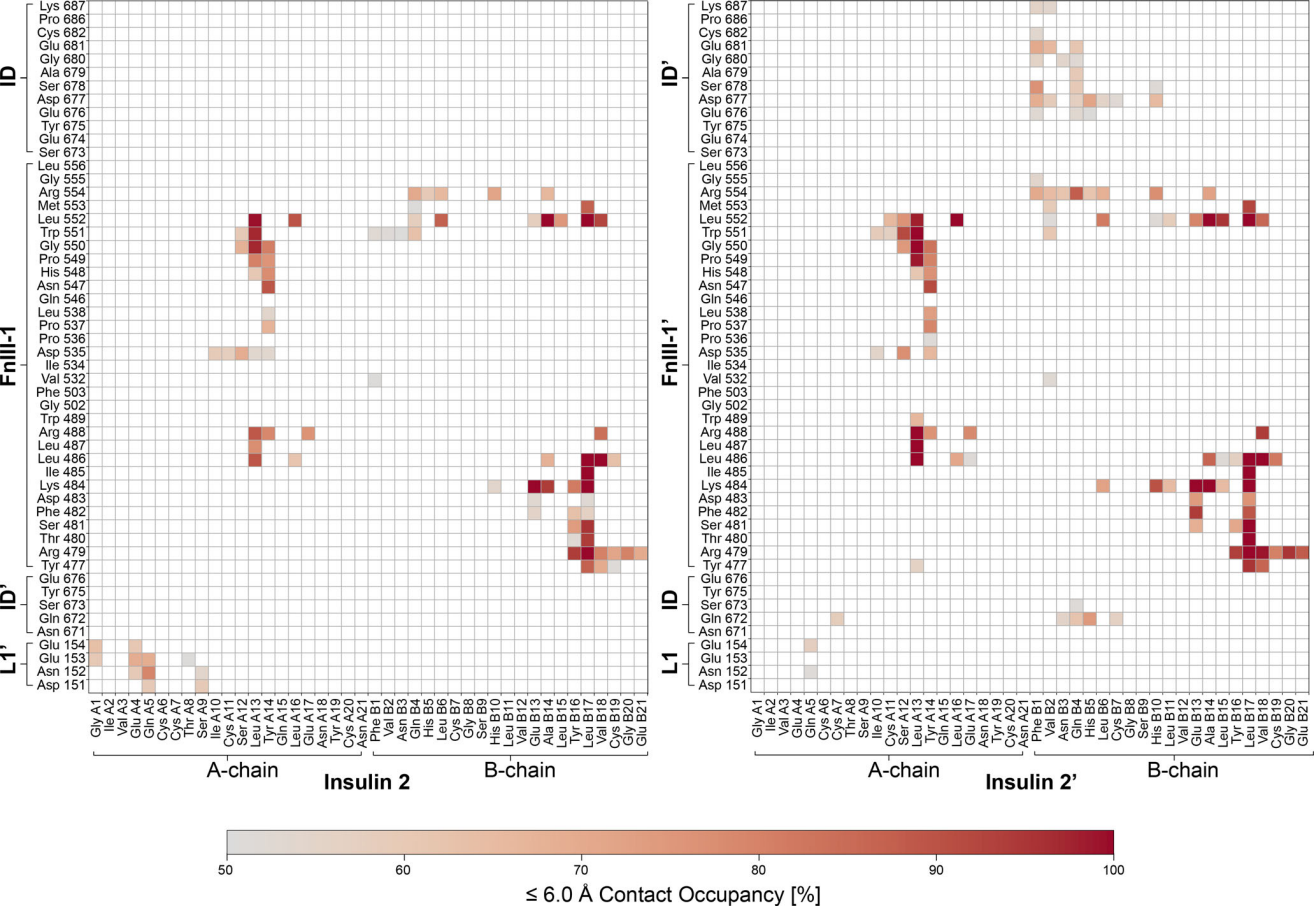
**Characterization of the novel insulin binding sites 2 and 2′ interactions by MD simulations.** Contact occupancies for insulin–IR-ECD interactions derived from our 10 × 500-ns MD simulations with a cutoff of 6.5 Å are depicted for insulin 2 (left) and insulin 2′ (right). Only contacts with an occupancy of ≥50% are displayed. See Figs. S5, S6, and S7 for contact maps comparing all insulin ligands in our cryo-EM structure and MD simulations.

**Figure 6.**
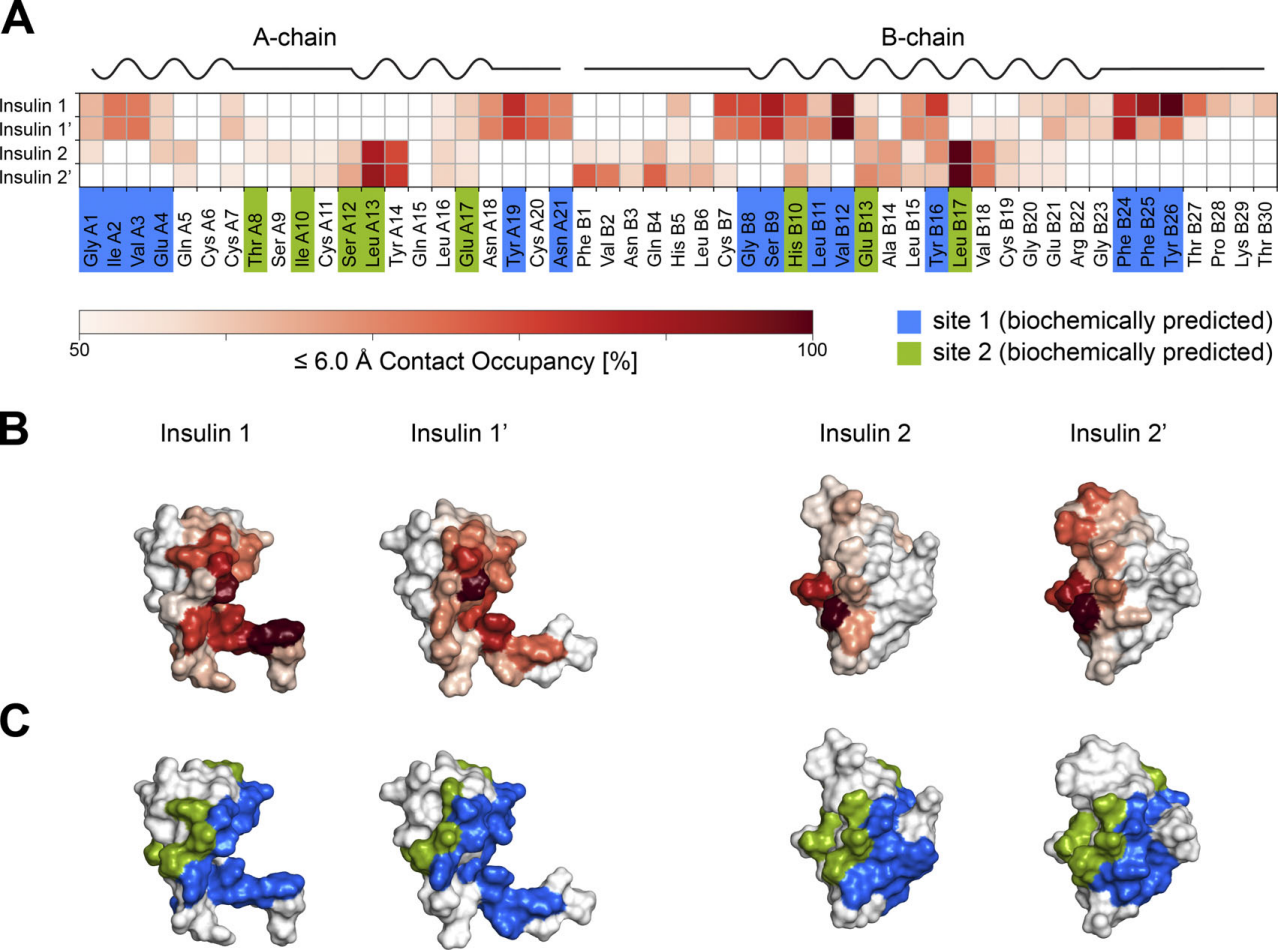
**Interactions of insulin with the IR-ECD binding sites 1 and 2.** **(A)** Summary of per residue contact occupancies of the four insulins bound to IR-ECD in the MD simulations. The contact occupancies are encoded by shades of red. Only contacts of ≥50% occupancy are displayed. Residues shown previously to contribute to interactions with IR-ECD site 1 and site 2 in biochemical experiments are highlighted in blue and green, respectively. The secondary structure of human insulin is drawn schematically above the contact map (based on PDB 3I3Z). **(B)** Contact occupancies color-coded as in A are plotted on the structural models of the four insulins bound to the IR-ECD. **(C)** This is juxtaposed with coloring of biochemically predicted site 1 and site 2 residues in blue and green, respectively.

**Figure S6.**
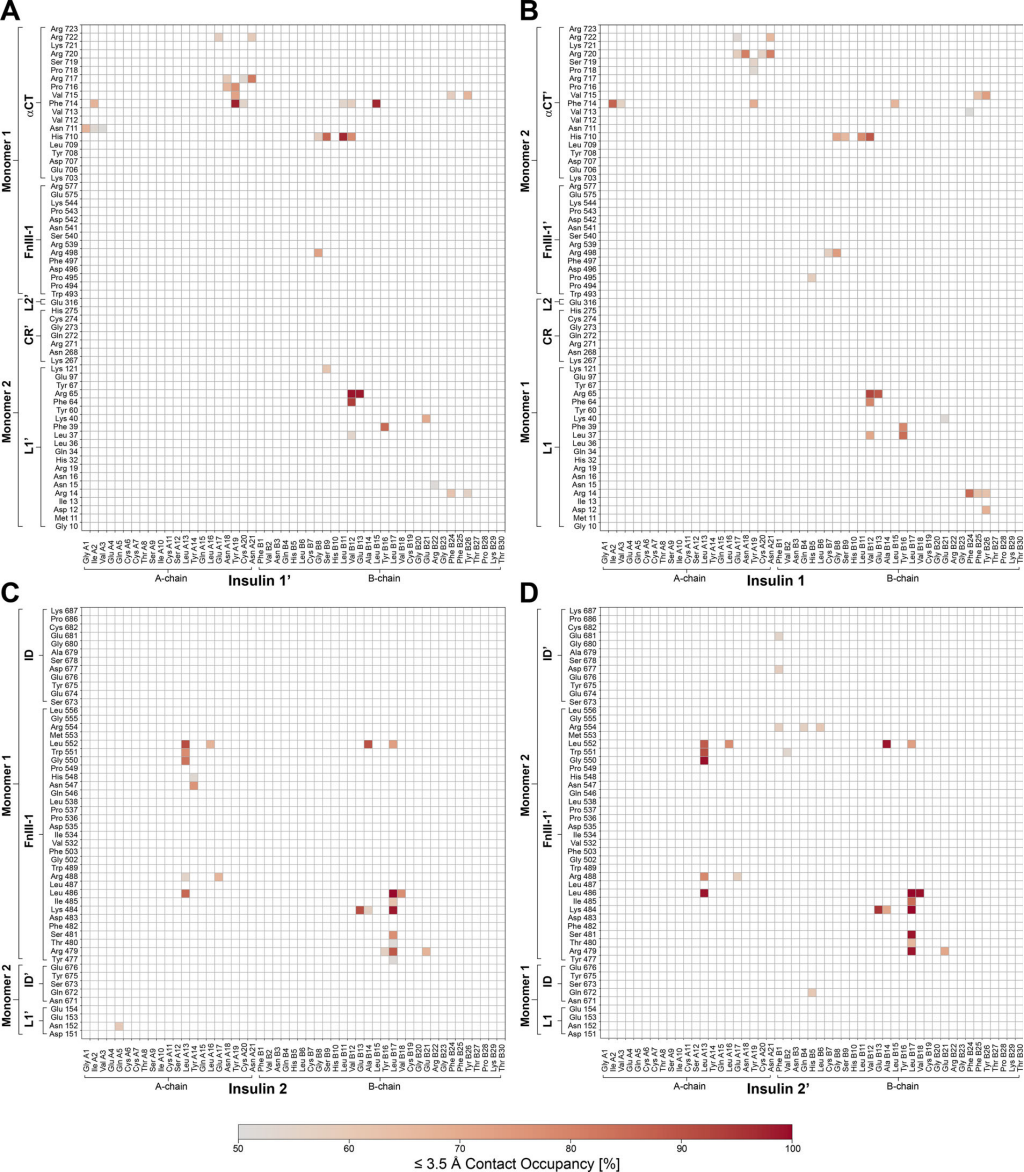
**Contact occupancies for insulin–IR-ECD interactions with a cutoff of 3.5 Å in the MD simulations.** **(A–D)** Contact map showing interactions between IR-ECD and head-bound insulins 1′/1 (A and B) and stalk-bound insulins 2/2′ (C and D). Only contacts with an occupancy >50% are displayed. The arrangement of the maps corresponds to the location of the respective insulin in the ECD (front view; Fig. 2 A).

**Figure S7.**
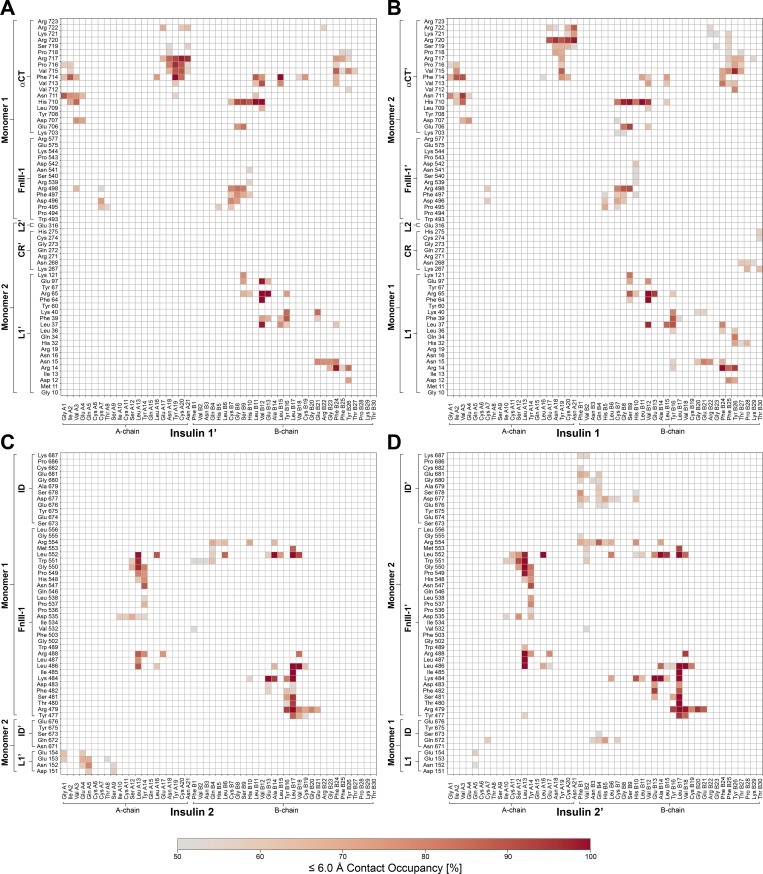
**Contact occupancies for insulin–IR-ECD interactions with a cutoff of 6.0 Å from MD simulations.** **(A–D)** Contact map showing interactions between IR-ECD and head-bound insulins 1′/1 (A and B) and stalk-bound insulins 2/2′ (C and D). Only contacts with an occupancy >50% are displayed. The arrangement of the maps corresponds to the location of the respective insulin in the ECD (front view).

The two newly identified insulins 2 and 2′ remained bound to the receptor during all 10 independent simulations of 500 ns each (Fig. 5; for direct comparison with insulins 1 and 1′, see Figs. S6 and S7). Three key areas of interaction stabilizing insulin 2 (or 2′) could be discerned. First, interactions formed between the central A-chain insulin residues (Ile A10–Glu A17) and FnIII-1 domain residues (Leu486–Arg488, Asp535–Leu538, and Asn547–Leu552); second, the interactions between N-terminal residues of the insulin B-chain (Phe B1–Leu B6) and FnIII-1 domain residues (Trp551–Arg554); and third, those between B-chain α-helix residues (His B10–Glu B21) and FnIII-1 domain residues (Tyr477–Arg488 and Leu552–Arg554). Interestingly, insulins 2 and 2′ display some asymmetry in their binding (Figs. 5 and 6). In particular, insulin 2′ appears to additionally interact with both ID loops (Gln 672–Ser673 and Glu676′–Cys682′). Insulin 2′–ID interactions were preserved across all 10 MD simulations. Moreover, insulin 2 appears to interact with its residues (Gly A1, Glu A4, Gln A5, Thr A8, and Ser A9) with a single L1′ domain loop (Asp151′–Glu154′). In the case of insulin 2′, only Gln A5 is in contact with L1 (Asn152 and Glu154). Stalk insulins 2/2′ mainly pack against β-strands of FnIII-1/1′ domains, which provide a stable interface for binding. In the case of head-bound insulins, the interaction site is composed of flexible loops from several domains. Thus, the binding of insulin 1 and 1′ depends on the proper organization of all required domains and is hence more sensitive to conformational variations.

Fluctuations within the insulin conformations during our simulations are mostly attributed to the flexible A- and B-chain termini, as indicated in the RMSD plots (Fig. S4, C and D). In particular, the insulin B-chain nonhelical N and C termini featured notable flexibility in our simulations (Fig. S4 D). C-terminal B-chain dynamics were also measured based on center-of-mass distances between the Cα atoms of the C-terminal residues (Phe B24–Thr B30) and B-chain α-helix residues (Gly B8, Val B12, and Leu B15; Fig. S4 E and Table S4). The C-terminal B-chain fluctuations were most pronounced in insulins 2 and 2′, which, unlike insulins 1 and 1′, do not engage their B-chain C termini with the receptor.

## Discussion

**Figure 7.**
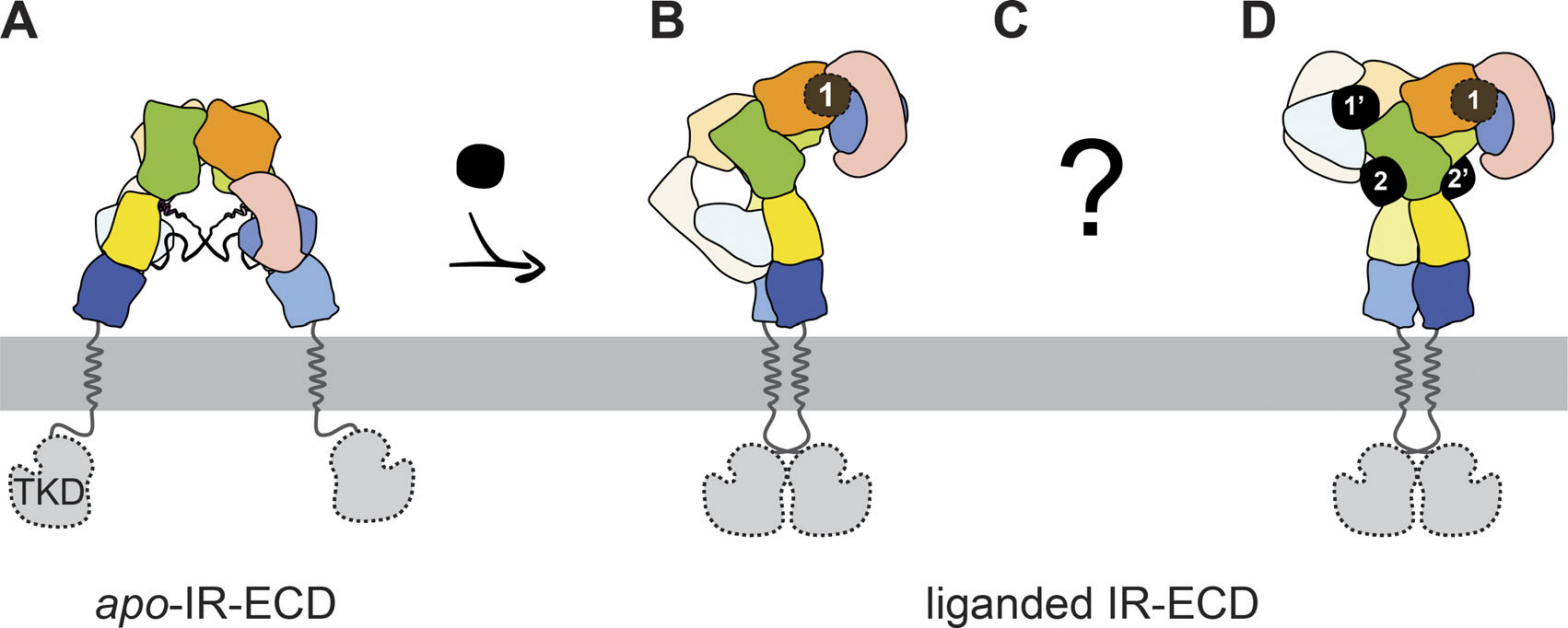
**Schematic models of unliganded and liganded transition states for which complete IR-ECD structures have been reported.** **(A)** *Apo*-IR-ECD (PDB 4ZXB; Croll et al., 2016). **(B)** Singly liganded IRΔβ-zipInsFv (PDB 6HN4 “lower” membrane-proximal part and PDB 6HN5 “upper” membrane-distal part; Weis et al., 2018). **(C)** Unknown intermediate states. **(D)** The four-insulin–saturated IR-ECD structure determined in this study. The subdomains are color-coded as in Fig. 1, and insulins are depicted in black with the respective binding site indicated.

Our cryo-EM structure of the human IR-ECD in complex with four insulin molecules offers new insights into the full IR-ECD in its ligand-saturated state and helps to reconcile a number of earlier findings to inform an integrated model of insulin–IR binding and activation (Fig. 7). Insulin binding to the full-length receptor is characterized by high- and low-affinity binding and/or negative cooperativity (De Meyts, 1994; Whittaker et al., 2008). Based on photo-cross-linking and mutagenesis screens, two distinct molecular surfaces on the insulin molecule have been identified to interact with two distinct receptor sites (1/1′ and 2/2′; De Meyts, 1994, 2015). It was furthermore proposed that insulin cross-links both receptor monomers by binding in a bivalent manner (De Meyts, 1994, 2015). The interactions of insulin with site 1 (and 1′) on the IR-ECD agree very well with previously described structures of insulin bound to the classic binding site 1/1′ (Menting et al., 2013, 2014; Scapin et al., 2018; Weis et al., 2018). The classic insulin site 1 residues, which are widely conserved during vertebrate evolution, participated in this interaction in our structure and in MD simulations (i.e., Gly A1–Glu A4, Tyr A19, Asn A21, Gly B8, Ser B9, Leu B11, Val B12, Tyr B16, Phe B24, Phe B25, and Tyr B26; Figs. 6, S5, S6, and S7). Insulin bound to site 1 (or 1′) simultaneously interacts with residues from the FnIII-1′ (or FnIII-1) domains, similar to the very recent IR-ECD cryo-EM structures (Scapin et al., 2018; Weis et al., 2018), supporting the bivalent insulin binding mode previously proposed (De Meyts, 1994; Schäffer, 1994). The structural basis for site 2 insulin interactions, however, remained elusive, and an irreconcilable difference between biochemical studies and previous cryo-EM data for insulin–IR engagement persisted.

The biochemically mapped sites 2 and 2′ involve residues in the IR-ECD FnIII-1 domains (De Meyts et al., 1978; Whittaker et al., 2008; De Meyts, 2015; Ye et al., 2017), which agree well with our structural data. We could not confirm the site 2/2′ residues that were proposed based on an amino acid residue conservation analysis and the reconstruction of the phylogeny of the IR family (Rentería et al., 2008). All insulin residues proposed to be involved in site 2 contacts participated in this interaction (i.e., Thr A8, Ile A10, Ser A12, Leu A13, Glu A17, His B10, Glu B13, and Leu B17; Fig. 6).

### Functional implications of site 2 and 2′ interactions with insulin

While the stalk-bound insulin molecules in our structure display a closed conformation reminiscent of insulin conformation in solution before binding (Hua et al., 1991), the head-bound insulins adopt an open conformation, previously described for receptor site 1 binding (Xu et al., 2009; Menting et al., 2014; Weiss and Lawrence, 2018). This suggests that site 2 (or 2′) interactions might be important for establishing the initial ligand–receptor contact and possibly contribute to ligand specificity, as previously discussed (De Meyts, 2004; Weis et al., 2018; Xu et al., 2018). The arrangement of sites 1 and 2′ in the *apo*-IR-ECD crystal structure also implicates sites 2 and 2′ as the sites of first contact. In this structure, sites 2 and 2′ are exposed and solvent accessible, while residues in sites 1 and 1′ appear partially engaged in interactions with the opposite FnIII-2 domain (McKern et al., 2006; Whittaker et al., 2008; Croll et al., 2016). Earlier biochemical findings had already indicated site 2 as the initial insulin contact site (De Meyts, 2004). Evolutionarily ancient vertebrate insulin from hagfish (*Myxine glutinosa*) was shown to exhibit anomalous binding behavior, different from most mammalian insulins. Despite absolute conservation of all site 1 residues and structural homology, hagfish insulin displays slow association kinetics, low affinity, low metabolic potency, and decreased negative cooperativity (Muggeo et al., 1979; De Meyts, 2004). This was attributed to variations in site 2 residues Leu A13 and Leu B17, which contribute significantly to site 2 interactions in our data (Figs. 5 and 6). Analogously, insulin variants carrying alanine replacements of those residues display a 20-fold decrease in initial ligand–receptor association (DeMeyts et al., 1976; De Meyts and Whittaker, 2002).

Based on the above-described structural and biochemical findings by us and others, a molecular model is envisaged where insulin initially interacts transiently with either of the exposed sites 2 or 2′. This may destabilize certain *apo*-conformation–specific, inhibitory domain–domain interactions such as the L1:FnIII-2′ cross-talk, which in turn permits T-shape specific domain–domain interactions. The resulting transition states may be similar to those captured in the cryo-EM data for unliganded IR-ECD (Fig. S1 I). The initial capture of insulin by site 2 may also relate to a conformational change important for ligand engagement with site 1. Indeed, the C-terminal segments of the insulin B-chains in both insulins 2 and 2′ were not involved in site 2 interactions at any time in our MD simulations, but seemed to sample their environment comparatively unrestrained. In a situation where site 2′ is liganded and site 1 is still in a transition state, these C termini might be free to establish contacts with site 1 adopting an open conformation. The possibility that these segments play a critical role in initiating a series of conformational changes was suggested very recently (Weiss and Lawrence, 2018). Finally, the receptor binding sites 1 and/or 1′ on the head are fully formed, and insulin interacts with both receptor monomers in a bivalent manner (De Meyts, 1994, 2015).

Micromolar insulin concentrations reverse negative cooperativity in the full-length cell surface receptor, and all unoccupied binding sites are believed to become saturated, inducing the so-called slowly dissociating (*K*_super_) state (de Meyts et al., 1973; De Meyts and Whittaker, 2002; Kiselyov et al., 2009). Under these conditions, site 2–bound insulins may assume yet another function by acting as a molecular wedge to prevent L1 from folding back onto FnIII-1/2′, thus stabilizing the T-shaped conformation. This view is also supported by our observation of L1/L1′ interacting with insulin 2′/2. Strikingly, the stalk-binding sites are partially asymmetric in our MD simulations: only one insulin molecule (insulin 2′ bound to FnIII-1′) appears to interact with residues of the ID regions of both monomers. It is therefore tempting to speculate whether this asymmetry-inducing interaction is critical for generating negative cooperativity in the cell surface receptor. Such an interaction would likely influence the positioning of the αCT/αCT′ helices critical for high-affinity binding and for cross-talk with the stalks. Another potentially cross-talking element might be the FnIII-1 domains, which feature one of the interprotomer disulfide bonds and contribute to binding of insulin to sites 1/1′ and 2/2′ (Fig. 4).

It is not known whether initial insulin docking at the receptor induces a conformational change or whether the receptor transiently adopts various conformations allowing the ligand to engage, or both. The high structural heterogeneity of the unliganded ECD observed here (Fig. S1 I) supports the view that the insulin-free IR adopts various transition states and thereby samples its environment. Even though this interpretation is in line with earlier in silico predictions, the precise contribution of distinct receptor conformations to signaling remains to be understood in detail (Kiselyov et al., 2009). This is not to imply that the four-insulin–bound state reported here necessarily corresponds to the most prevalent, physiologically active IR conformation. However, this structure exhibits all possible binding sites, including those important for initial contact, albeit with possibly differing precise binding modes in the transition states (Fig. 7).

**Figure S8.**
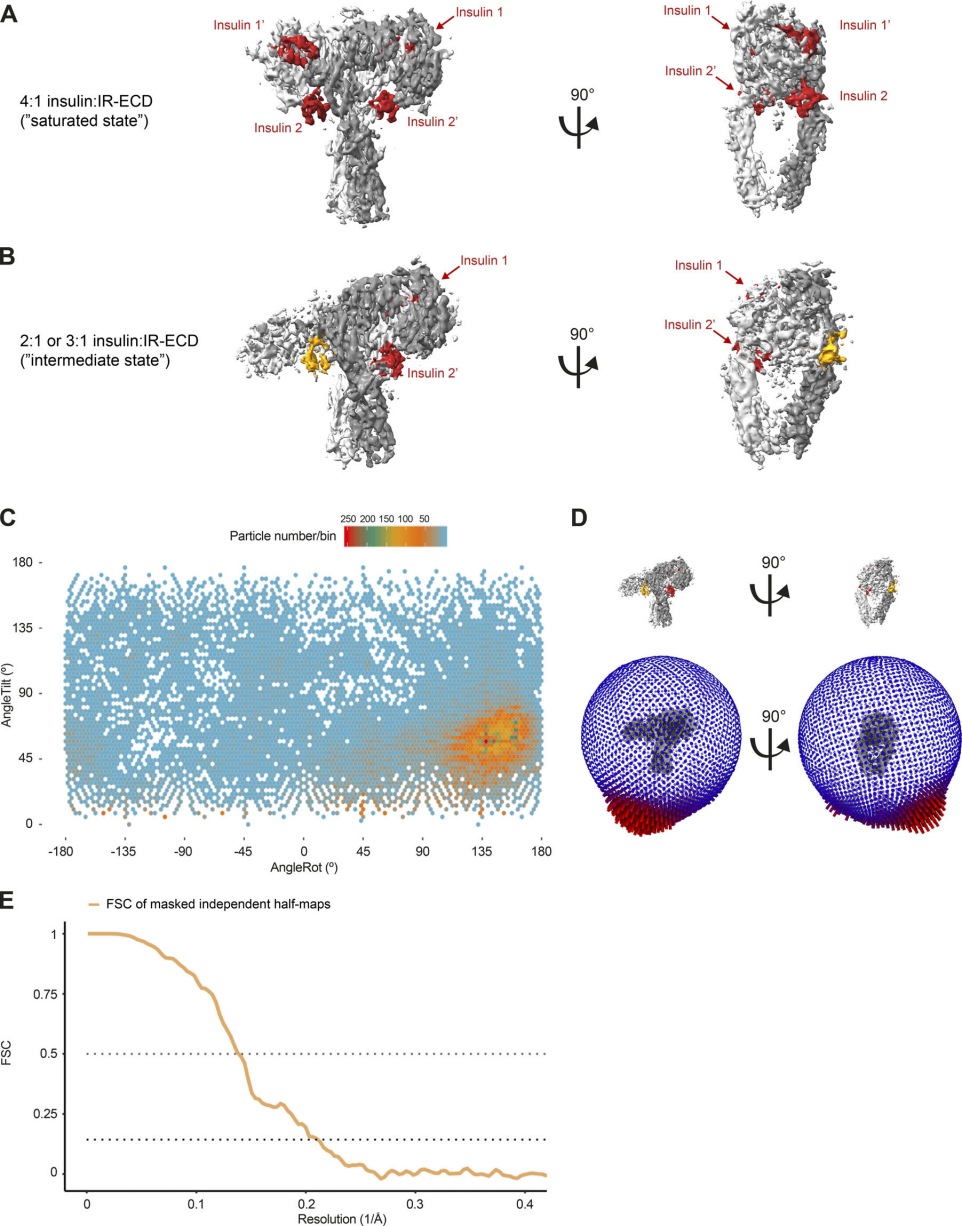
**Cryo-EM analysis of the insulin–IR-ECD intermediate state.** **(A and B)** Front and side views of the 3D density maps of the saturated state bound to four insulins (A) and the intermediate state bound to at least two insulins (B). Densities corresponding to the first and second IRαβ_0_ protomers are colored in gray and white, respectively. Insulin moieties are depicted in red. For our intermediate state reconstruction, we could unambiguously assign insulin 1 and 2′ (in red). A density juxtaposed to binding site 2 (yellow) might correspond to insulin, but is insufficiently resolved to exclude it from corresponding to the receptor, such as the αCT peptide. We cautiously assign the intermediate state reconstruction as a two- or three-insulin–bound structural intermediate. The domains in the receptor head lacking insulin are strikingly tilted, and the FnIII-3 domains converge. **(C)** Angular distribution of particles contributing to the intermediate state reconstruction. Tilt and rotation angles were plotted against each other for the final intermediate state 3D reconstruction. The color of each sampling bin indicates the number of particles in the respective bin. **(D)** In the spherical angular distribution representation, blue denotes fewer, and red more, particles (50,079 particles in total). AngleRot, rotation angle. **(E)** FSC of masked independent half-maps of the final intermediate state map. The nominal global resolution of the intermediate state reconstruction was 5.0 Å according to the 0.143 cutoff criterion (Rosenthal and Henderson, 2003). This is most likely an overestimate due to the anisotropy in the distribution of views. For ease of interpretation, both the 0.5 and 0.143 cutoffs are indicated by dotted lines.

Interestingly, we observed higher conformational fluctuations in insulin 1′ compared with insulin 1 in our MD simulations (Fig. S4 C). This behavior is also reflected in overall weaker C-terminal B-chain contacts between insulin 1′ and the receptor, which are critical for binding (Fig. 6). We then decided to reevaluate our cryo-EM dataset, which led to the identification of a second, minor 3D class with a potentially distinct ligand binding site occupancy (Fig. S2, “intermediate state”). This small, but stable, 3D class of particles captures a distinct conformation of the receptor with at least two insulins bound to sites 1 and 2′, with the membrane proximal FnIII-3 domains remaining converged (Fig. S8 B). The reconstruction is not as highly resolved compared with the four-insulin–bound state (5-Å global resolution estimate according to the 0.143 FSC cutoff criterion) and comprises merely 1.7% of the initial particles. Surprisingly, however, the domains in the receptor head lacking insulin are strikingly tilted. An additional density proximal to site 2 is consistent with a bound insulin in both size and shape, but is insufficiently resolved to exclude it being portions of the receptor (e.g., αCT peptide). As such, we cautiously assign the “intermediate state” reconstruction as a two- or three-insulin–bound structural intermediate. This second 3D reconstruction provides evidence that even at the saturating insulin concentration used here, the receptor adopts various liganded states.

### Ligand occupancy and implications for ligand selectivity

Our finding that the human IR binds up to four insulin molecules simultaneously (Fig. 7) provides a hint as to how ligand specificity and selectivity might be realized in the cellular context, considering that a single receptor exhibits distinct responses to different ligands. In humans, the relevant ligands are insulin-like growth factor 1 and 2, in addition to insulin. Further examples of ligand heterogeneity are the homologous invertebrate insulin-like receptors. *Drosophila melanogaster* expresses 7 insulin-like peptide ligands for a single insulin-like receptor, while *Caenorhabditis elegans* even has 40 insulin-like peptides that have been genetically identified and shown to act in a combinatorial manner to coordinate various physiological processes (Garofalo, 2002; Fernandes de Abreu et al., 2014). This diversity emphasizes how important careful examinations of even the subtlest asymmetries within the receptors are, as they likely are significant for high-affinity ligand binding, cooperativity, ligand selectivity, and signaling outcome, as shown for the EGF receptor (Alvarado et al., 2010; Freed et al., 2017). For this reason, we refrained from imposing any symmetry in our reconstructions, thereby allowing us to unequivocally identify asymmetries within binding pockets, the ID domains, and the fibronectin stalks, all of which are likely to constitute functional features of the receptor.

After depositing our manuscript on the four-insulin:IR-ECD structure to *bioRxiv* (Gutmann et al., 2019 *Preprint*) and during the revision of this manuscript, an additional cryo-EM study was published (Uchikawa et al., 2019). The authors reported a single structure of the ECD of a detergent-solubilized full-length IR in dodecylmaltoside micelles. By enforcing C2 symmetry, this reconstruction reached a global resolution estimated as 3.2 Å. The structure confirms the “saturated” T-shaped four-insulin–bound ECD structure seen here, especially with regard to the receptor head. However, the imposed C2 symmetry masks the intricacies of binding asymmetry that is evident in our map and simulations. Furthermore, our intermediate state map demonstrates that a change in occupancy is concomitant with a conformational change. In turn, this raises questions concerning the utility of symmetry expansion for examining ligand occupancy.

Future work is required to study the full-length receptor in a membrane context; in particular considering the well-documented detergent bias (varying with the detergent to protein ratio) on the receptor leading from hypersensitization to inhibition (Leray et al., 1992; Delle Bovi and Miller, 2017). Further concerns persist, as previous studies dating back to the 1990s reported Y- or T-like structures already for detergent-solubilized full-length IR, which did not display conformational changes upon insulin addition (Christiansen et al., 1991; Tranum-Jensen et al., 1994; Woldin et al., 1999). A C2-symmetrized reconstruction of a detergent-solubilized IR in complex with gold-labeled insulin determined by CryoSTEM, on the other hand, suggested an altogether different structure (Luo et al., 1999), which we cannot easily reconcile with our model and other recent structures (Scapin et al., 2018; Weis et al., 2018; Uchikawa et al., 2019). The in vitro reconstitution of full-length IR in artificial membrane systems and thorough detergent removal were key for capturing the IR in a ligand-sensitive *apo*-conformation, which underwent large rigid body rotations into the T-shape in an insulin dose-dependent manner (Gutmann et al., 2018).

### Outlook

Although insulin replacement remains an essential therapy, it is still hampered by the inability of exogenously administered insulins to recapitulate the full spectrum of physiological insulin action (Jiráček and Žáková, 2017). A thorough understanding of the molecular details of ligand–IR activation is a prerequisite for the development of specific agonists as well as antagonists. Ligand titration with insulin, other agonists, or antagonists will help to capture the initial insulin docking and various conformational transition states of the receptor. Furthermore, in light of recent structural insights, the current mathematical binding models for insulin binding require reevaluation. The MD system reported here provides a valuable resource for engineering and testing novel ligands in silico.

We, and others, have suggested that the transmembrane signaling mechanism of the IR relies on the control of the distance between the transmembrane domains exerted by its ECD (Whittaker et al., 1994; Kavran et al., 2014; Gutmann et al., 2018). Similar to the IR, the distance between the membrane-proximal regions of the structurally related mitogenic insulin-like growth factor-1 receptor ECD is reduced from ∼115 to ∼67 Å in its unliganded form (Xu et al., 2018). This implies that the fine tuning of transmembrane domain positioning and orientation may be an intricate detail of ligand selectivity and cell signaling outcomes via allosteric domain coupling across the membrane. Therefore, integrating membrane lipid composition and lipid–protein interactions at the next level of reconstruction and analysis will indispensably contribute to a complete understanding of receptor function (Coskun and Simons, 2011; Endres et al., 2014; Kaszuba et al., 2015) and improved pharmacological targeting.

## Materials and methods

### Cloning and production of IR-ECD

A gene encoding human IR-ECD (IR signal sequence followed by residues 1–917 of the mature IR isoform A; UniProt entry P06213-2) followed at its C terminus by the 25-residue sequence SSGPSGSHHHHHHHHGSLEVLFQGP (i.e., a protease-resistant linker, the 8xHis tag, and the human rhinovirus 3C protease cleavage site) and a tandem-affinity purification tag (Rigaut et al., 1999) was cloned into the pTT6 vector, called pTT6-IRA.ECD-8xHis-TAP, for transient expression in mammalian cells. The pTT6 vector, which was derived from pTT3 (Durocher et al., 2002), featuring a Kozak sequence and a modified multiple cloning site, was kindly provided by the Protein Expression Purification and Characterization facility at the Max Planck Institute of Molecular Cell Biology and Genetics, Dresden, Germany. FreeStyle HEK293F cells (R79007, Thermo Fisher Scientific; RRID:CVCL_D603) were maintained in suspension in protein-free, chemically defined FreeStyle 293 Expression Medium (R79007, Thermo Fisher Scientific) supplemented with 1× penicillin/streptavidin (15140122, Thermo Fisher Scientific) at 90 rpm, 8% CO_2_, 37°C. Before transfection, the medium was replaced with fresh antibiotic-free medium. 2 liters of FreeStyle HEK293F cells were transiently transfected with pTT6-IRA.ECD-8xHis-TAP at a density of 2 × 10^6^ cells/ml by transfection with 2 mg endotoxin-free DNA precomplexed with polyethylenimine (at a ratio of 5:1 wt/wt to DNA; Longo et al., 2013). Upon transfection, cells were maintained for 64 h at 31°C, 8% CO_2_, 90 rpm. The conditioned medium was harvested by pelleting the cells at 300 *g*, 10 min, 25°C. Cells could be maintained for three more days in fresh medium for a second round of purification.

### Affinity purification of IR-ECD

Human IR-ECD (i.e., IR(αβ_0_)_2_) was purified from a 2-liter batch of conditioned medium. The medium was cleared by centrifugation at 2,500 *g*, 10 min, 4°C, and the supernatant was then allowed to bind to 4 ml IgG Sepharose beads for 3 h at 4°C under constant agitation, and then loaded onto a 2.5 × 20-cm Econo-Column glass chromatography column (7372522, Bio-Rad Laboratories). The flow-through was collected and reloaded onto the column. Running buffers were all based on Hepes-buffered saline (HBS; 50 mM Hepes, pH 7.5, and 150 mM NaCl), and all purification steps were performed at 4°C. The IgG Sepharose beads were washed with 10 column volumes (CV) of running buffer (RB; 150 mM NaCl, 50 mM Hepes, pH 7.5, and 5% vol/vol glycerol), 2 CV RB-ATP (RB + 5 mM ATP and 10 mM MgCl_2_), 2 CV RB-EDTA (RB + 20 mM EDTA), and then 20 CV elution buffer (RB + 15% vol/vol glycerol). For elution, IgG beads were incubated with glutathione S-transferase–tagged human rhinovirus 3C protease (50 µg protease per ml beads; provided by the Max Planck Institute of Molecular Cell Biology and Genetics) for TAP tag cleavage overnight at 4°C. After protease cleavage, IR-ECD was eluted in one step with 2.5 CV elution buffer. To remove coeluting protease and other impurities, the eluate was incubated with 1 ml Ni-NTA Superflow beads (Qiagen, 30430) for 3 h at 4°C on a rotating wheel for immobilized metal ion affinity chromatography (IMAC). The slurry of resin was then loaded onto a disposable conical 0.8 × 4-cm polypropylene column (Bio-Rad Laboratories). The flow-through was collected and reloaded onto the column. The resin was washed with 10 CV HBS including 10 mM imidazole (3899.3, Carl Roth) and eluted with HBS including 280 mM imidazole in 1-CV fractions. The pH of the wash and elution buffers was adjusted to 7.5. IMAC elution fractions were analyzed by reducing SDS-PAGE using precast NuPAGE 4–12% Bis-Tris gels (Thermo Fisher Scientific) with 1× MOPS buffer (Thermo Fisher Scientific) and subsequent Coomassie Brilliant Blue G-250 staining (Fig. S1 A).

For biochemical studies, IMAC elution fractions were directly subjected to gel filtration using a Superdex 200 Increase 10/300 GL column equilibrated in HBS at a flow rate of 0.5 ml/min at room temperature to separate dimeric IR-ECD from monomeric IRαβ_0_. The peak fraction containing IR-ECD was stored at 4°C until further use, within 72 h. The apparent molecular weight of IR-ECD was estimated in SDS-PAGE with 3–8% Tris-Acetate gels (Life Technologies) using HiMark unstained protein standards (Thermo Fisher Scientific; Fig. S1). The IR-ECD concentration was estimated using a molar extinction coefficient for IR(αβ_0_)_2_ of 280,260 M^−1^ cm^−1^ at 280 nm absorbance (as calculated by ExPASy/ProtParam [Gasteiger et al., 2003] assuming one free thiol group per monomer [Chiacchia, 1991; Sparrow et al., 1997]). This concentration estimate was confirmed once with a BCA protein assay (Thermo Fisher Scientific).

For cryo-EM studies, IMAC elution fractions containing most concentrated IR-ECD were immediately desalted after immobilized metal affinity chromatography elution using disposable 8.3-ml Sephadex G-25 PD-10 desalting columns, concentrated to 3 µM in Amicon Ultra-0.5 ml ultrafiltration units with Ultracel-100 membranes (Merck Chemicals), and kept on ice until further use within 24 h. The concentrated protein was gel-filtrated using a Superdex 200 10/300 GL column equilibrated in HBS at a flow rate of 0.5 ml/min and 4°C (GE Healthcare). The peak fraction containing IR-ECD was immediately used for cryo-EM sample preparation.

### MST to determine insulin binding to IR-ECD

Recombinant human insulin was purchased from Sigma-Aldrich (I2643, lot SLBR9404V, expressed in yeast, 99% purity by HPLC, 0.4% zinc) and resuspended in 5 mM HCl at 3 mg/ml (20252.244, VWR Chemicals). The purity of insulin was confirmed by mass spectrometry under denaturing conditions where only monomeric and dimeric insulin with a mass of 5803.651 ± 0.003 Daltons was detectable, corresponding to the expected mass of insulin with all three disulfide bridges formed. Under native conditions, as expected, additional peaks corresponding to higher insulin oligomers appeared.

Insulin binding to IR-ECD was analyzed by MST. First, IR-ECD was diluted to a final concentration of 100 nM in HBS-T (HBS, pH 7.5, and 0.05% Tween-20) and labeled with 25 nM tris-nitriloacetic acid conjugated to NT647 (red tris-NTA; Lata et al., 2005; Bartoschik et al., 2018), which was a kind gift of Jacob Piehler (University Osnabrück, Osnabrück, Germany). A label-to-IR(αβ_0_)_2_ molar ratio of 1:4 was chosen to circumvent interference of free dye. The reaction was incubated for 30 min in the dark at room temperature and was subsequently centrifuged at 14,000 *g* for 10 min at 4°C.

For ligand binding assays, a 100-µM stock solution of recombinant human insulin in 5 mM HCl was diluted to a concentration of 5 µM in HBS-T. A serial dilution was prepared with ligand binding buffer (i.e., HBS with 25 µM HCl). 10 µl of the diluted ligand was incubated with 10 µl of 20 nM IR-ECD overnight at 4°C. Thus, the final assay concentrations were 10 nM IR-ECD and 2.5 nM red tris-NTA.

MST was performed in standard capillaries (MO-K022, Nanotemper Technologies) on a Monolith NT.115^Pico^ instrument (Nanotemper Technologies) using the Pico-RED detector with 30% light-emitting diode power at 25°C. Data were analyzed with MO.Affinity Analysis 2.2.7 software (NanoTemper Technologies). The integral of thermophoresis traces from 1 to 20 s on-time was used for binding affinity determination, and the normalized fluorescence difference ΔF_norm_ was plotted against ligand concentration for dose–response plots. To determine *K*_d_ values, nonlinear regression (one-site binding) was performed using Prism version 7.0 for Windows (GraphPad Software).

A concentration range, within which insulin did not appear to interact nonspecifically with the labeling reaction or with the dye itself, was determined by titrating insulin against a red tris-NTA–labeled control peptide (comprising an 8xHis tag and part of the HRV3C cleavage site, i.e., _2_HN-HHHHHHHHKLEVLF-CONH_2_).

### Thermal stability by nano–differential scanning fluorimetry (nanoDSF)

To further characterize IR-ECD and to monitor its stability, a thermal unfolding assay was performed applying label-free, low-volume nanoDSF. IR-ECD was diluted to 500 nM in HBS and incubated with or without 50 µM insulin for 1 h on ice in a volume of 22 µl. Samples were loaded into nanoDSF Grade Standard capillaries (PR-C002, NanoTemper Technologies) in duplicate and transferred to a Prometheus NT.48 instrument (NanoTemper Technologies). Thermal unfolding was detected by recording the intrinsic tryptophan fluorescence (emission ratio at 350 and 330 nm) during heating in a linear thermal ramp (1°C/min; 20°C to 95°C) with an excitation power of 100%.

### Cryo-EM grid preparation and imaging

Peak fractions at a final Abs_280nm_ of ∼0.4 (∼1.4 µM) were incubated for ∼30 min at 4°C with or without recombinant human insulin supplementation at 28× molar excess (∼40 µM final concentration). 4 µl of these samples were applied to glow discharged (2.2 × 10^−1^ mbar for 2 × 20 s) Quantifoil holey carbon grids (R2/1, 200 mesh, Quantifoil). The grids were plunge vitrified in a liquid ethane/propane mix using a Vitrobot Mark IV at 4°C and 95% humidity. Cryo-EM data were collected on a FEI Titan Krios microscope operated at 300 kV, equipped with a postcolumn Gatan energy filter and a K2 Summit direct detector operating in counting mode. A total of 8,882 movies were recorded at a nominal magnification of 130,000× that corresponds to 1.059 Å/pixel at the specimen level using SerialEM (Mastronarde, 2005). The total exposure of 55 e^−^/Å^2^ at the specimen level was evenly distributed over 51 frames during 10.2 s. The preset target defocus range was 0.5–3.5 µm. The sample preparation and data collection strategies for the *apo*-IR-ECD samples were very similar except that no insulin was used for grid preparation. These data were collected with a total exposure of 59 e^−^/Å^2^, spread over 51 frames and 10.2 s. The target defocus ranged from 0.5 to 3.5 µm. No stage pretilt was used for either of the two datasets.

### Cryo-EM data acquisition and processing

The RELION-3.0 implementation of MotionCor2 (Zheng et al., 2017) was used to correct for beam-induced sample motions and radiation damage. The summed and dose-weighted micrographs were used for further processing. Particles were selected using Gautomatch version 0.56 (https://www.mrc-lmb.cam.ac.uk/kzhang/Gautomatch/). CTF parameters were determined using Gctf (Zhang, 2016). If not stated otherwise, all further processing was performed in RELION v2.1 or v3.0 (Kimanius et al., 2016; Zivanov et al., 2018). In the case of the insulin-bound structure, initial analysis of particles picked without templates yielded a 3D reconstruction using as template a 60-Å low-pass filtered initial model generated by the stochastic gradient descent implementation of RELION v2.1 (Kimanius et al., 2016; compare Fig. S2 for a graphical overview of the processing routine). Low-pass filtered projections of this reconstruction were used as templates for template-based particle picking on all micrographs. This resulted in 2,997,079 particle candidates. The particle stack was cleaned up by unsupervised 2D classification in subsets of ∼120,000 particles. Subsequently, the data were further processed in ∼120,000 particle chunks in 3D classification with the first reconstruction as a 60-Å low-pass filtered starting model. The resulting cleaned dataset of 326,257 particles reached a nominal global resolution of 4.9 Å after 3D refinement and postprocessing. Bayesian polishing in RELION v3.0 (Zivanov et al., 2018) was used to correct further for beam-induced motion and radiation damage, improving the quality of the map to a final apparent resolution of 4.3 Å. The global resolution estimates of the obtained reconstructions are quoted as good proxies for the overall relative quality of the individual reconstructions, fully acknowledging the differences in local resolution estimates as well as the anisotropy of the data. The angular distribution of particles contributing to this map is shown in Fig. S3, C and D, and the FSC curve of the masked independent half-maps in Fig. S3 E. The rotation versus tilt angle plot in Fig. S3 C was created by binning the angular assignments of all particles contributing to this reconstruction in 3° × 1.5° bins, followed by plotting the resulting distribution using the Tidyverse collection of R packages (https://www.tidyverse.org/). The local resolution estimate in Fig. S3 F was calculated with the local resolution routine implemented in RELION v3.0 (Zivanov et al., 2018).

The minor class we termed “intermediate state,” which is described in Figs. S2 and S8, was processed in a similar way as the “saturated state.” After the above-described classification in ∼120,000 particle chunks, 50,079 particles were subjected to 3D refinement focused on the ECD “head.” This reconstruction reached a nominal global resolution of 5.0 Å (according to the 0.143 FSC cutoff criterion) after Bayesian polishing in RELION v3.0 (Zivanov et al., 2018), a further 3D refinement step of the whole ECD as well as map filtering and sharpening. The rotation versus tilt angle plot in Fig. S8 C was created by binning the angular assignments of all particles contributing to this reconstruction in 3° × 3° bins, followed by plotting the resulting distribution using the Tidyverse collection of R packages.

The data of the ligand-free IR-ECD sample were processed using an approach similar to that outlined above. Since the attempts at 3D reconstruction never yielded resolutions in the subnanometer range, only 2D class averages are shown.

### Model building and refinement

A nonglycosylated IR-ECD model with four insulins was constructed initially for fitting into the cryo-EM density map. This model was based on the previously published partial insulin-bound IR-ECD (PDB 6CEB, including the two head-bound insulins 1 and 1′). The L1 and L1′ domain residues H144 were modified to Y144 to match the IR construct used here (Uniprot P06213-2). Regions that were not resolved in PDB 6CEB were added as described in the following. As reliable starting models for the FnIII domains, we included the respective coordinates from PDB 4ZXB (Croll et al., 2016) in the structure. Additionally, we constructed tentative models of the IDα loops (chain α and α′ residues 651–687) using MODELLER (Eswar et al., 2006) and included them in the structure, since we observed some incohesive density features for these regions. The stalk-bound insulins 2 and 2′ were modeled based on the structure of porcine insulin (PDB 4INS; Baker et al., 1988). To match the human insulin sequence, the insulin B-chain C terminus residue was mutated from A30 to T30. The model is thus consistent with the complete human IR-ECD (UniProt P06213-2) and human insulin sequences (UniProt P01308) and matches the experimental constructs used in this study.

As a first step in the fitting procedure, global, rigid body docking of the resulting nonglycosylated IR-ECD in complex with four insulins into the density map was performed in UCSF Chimera (Pettersen et al., 2004). To locally improve the model, we used a combination of flexible fitting methods including the real-space structure refinement program DireX (Wang and Schröder, 2012), followed by the simple relax protocol in torsional space in Rosetta (Fleishman et al., 2011; Conway et al., 2014). As a last step, the cysteines involved in intra- and interchain dimer bonds as well as specific β-strands of the FnIII-3 domains were directed into selected regions of the density map by interactive MD flexible fitting (Trabuco et al., 2009; McGreevy et al., 2016).

After completion of the initial fitting routine outlined above, the structure was subjected to several rounds of iterative real-space refinement in phenix.refine (Afonine et al., 2018) and manual adjustment in Coot (Emsley et al., 2010). Progress in modeling was monitored via the map-to-model correlation coefficients, geometry indicators, and the map-versus-model FSC (see Table S1). Structure images were created in PyMOL2 (PyMOL Molecular Graphiscs System, Schrödinger) and ChimeraX (Goddard et al., 2018). The refined model is deposited in PDB (accession number 6SOF) and is referred to in the main text as “cryo-EM structure.”

Since our reconstruction did not produce clear density features for the Arg-Lys-Arg-Arg residues (furin cleavage site), the disordered IDβ region, and the residual C-terminal purification tag sequence, these parts are not included in the refined structure (see also Table S2). However, for completeness, they are included in the model used in MD simulations described below.

We decided to include the modeled side chains in our IR-ECD structure bound to four insulins (PDB 6CEB), since it is our belief that the resulting model most closely approximates our experimental cryo-EM data. In our view, this is justified because portions of the reconstruction are resolved to ∼4 Å (especially the core of the head domain; see local resolution estimate in Fig. S3 F), at which point individual bulky side chains become discernible. In addition, even at lower resolution, side chains potentially contribute to the signal in the particle images. This is supported by the model-to-map correlation coefficients being lower in the absence of side chains compared with the deposited model (e.g., CC_mask_ of 0.63 for no-side chains vs. 0.72 with side chains model). However, we strongly advise readers against interpreting side chain–level interactions in our model, as there is insufficient basis for such interpretations from our cryo-EM density.

### Atomistic MD simulations

For atomistic MD simulations, we completed the structure refined against the EM map described above by adding all the loops invisible in our density map (i.e., the furin cleavage site, residues 720–723), the disordered and highly glycosylated N-terminal region of the IRβ subunit (residues 724–756), and the residual C-terminal purification tag sequence (SSGPSGSHHHHHHHHGSLEVLFQ). All of these additional loops and regions were built using MODELLER (Eswar et al., 2006). Additionally, based on the glycan composition defined previously (Sparrow et al., 2007, 2008), we added 17 *N*-linked and 6 *O*-linked glycans on each monomer (Table S3) using the doGlycans tool (Danne et al., 2017). The OPLS-AA force field (Kaminski et al., 2001; Danne et al., 2017) was used for proteins, glycans, and ions. The glycosylated IR-ECD was energy-minimized in vacuum using the steepest descent algorithm to remove any steric clashes due to overlapping atoms. The energy-minimized structure was then solvated using the TIP3P water model (Jorgensen et al., 1983) in a box of 21 nm^3^. The solvated structure was neutralized with an appropriate number of Na^+^ counterions complemented by 150 mM NaCl to match experimental buffer and salt concentration. The system consisted of 924,775 atoms in total. The resulting structural model of the IR-ECD is referred to as “MD model” in the text.

Before MD simulations, the system was again subjected to energy minimization followed by 50-ns equilibration under NVT (constant particle number, volume, and temperature) conditions at 298 K using the v-rescale thermostat (Bussi et al., 2007) with a time constant of 0.1 ps. At this stage, the IR-ECD and the insulin backbone atoms were position-restrained with a force constant of 1,000 kJ mol^−1^ nm^−2^. Next, equilibration of the system was continued under NpT (constant particle number, pressure, and temperature) conditions using isotropic Parrinello–Rahman pressure coupling (Parrinello and Rahman, 1980, 1981, 1982) with a time constant of 2 ps over a period of 50 ns, with reference pressure set to 1 bar and isothermal compressibility to 4.5 × 10^−5^ bar^−1^. The IR-ECD and the insulin backbone atoms were again position-restrained with a force constant of 500 kJ mol^−1^ nm^−2^. The resulting structure is referred to as “starting MD model” throughout the text. Electrostatic interactions were calculated by the particle mesh Ewald method (Darden et al., 1993; Essmann et al., 1995) using 1.0 nm for the cutoff of the real space component. The same cutoff distance was set for van der Waals interactions together with the LINCS algorithm (Hess et al., 1997) for all bonds. Periodic boundary conditions were applied in all three dimensions. The final production run for 500 ns was performed after removal of all position restraints, and the rest of the input parameters were the same as those used under NpT equilibration simulations. All MD simulations were performed with an integration time step of 2 fs using the GROMACS 4.6 simulation package (Hess et al., 2008), and the output trajectory and energies were saved every 100 ps. For reproducibility of the results, 10 repeats (500 ns each) were performed.

For the analyses, GROMACS tools and in-house built scripts were used. Contact maps were built with the g_distMat analysis tool. A contact for a given pair of residues was considered to be established if the minimum distance between any atoms in the two residues was either ≤3.5 Å or ≤6 Å. RMSD analysis was performed for backbone atoms with respect to the starting MD model. The final 100 ns from each of the 10 trajectories were used to generate residue contact occupancy maps. MD movies and figures were prepared using VMD (Humphrey et al., 1996) and PyMOL2.

### Data availability

The cryo-EM density maps and structural model of the 4:1 insulin–IR-ECD complex developed in this study are available from the Electron Microscopy Data Bank (EMD-10273) and Protein Data Bank (PDB 6SOF), respectively. The cryo-EM density map for the IR-ECD bound by several insulins in an intermediate state is available from the Electron Microscopy Data Bank (EMD-10311).

### Online supplemental material

Fig. S1 illustrates the IR-ECD purification, the analysis of insulin binding, and 2D class averages of *apo*-IR-ECD. Figs. S2 and S3 give an overview on cryo-EM data collection and processing. Fig. S4 demonstrates asymmetries in our cryo-EM structure and flexibilities or fluctuations of IR-ECD and its insulin ligands during our MD simulations. Figs. S5, S6, and S7 provide contact and occupancy maps based on the cryo-EM structure or MD simulations. Fig. S8 gives an overview of the cryo-EM data processing for our 3D reconstruction of the intermediate state IR-ECD bound to several insulins. Table S1 provides a summary of the cryo-EM data collection and model quality indicators. Table S2 summarizes all residues included or absent from the insulin–IR-ECD cryo-EM structure. The glycan composition of IR-ECD in our MD simulation model is in Table S3. Table S4 provides center-of-mass distance measurements between insulin B-chain C-terminal and B-chain α-helix residues for our 10-MD simulations.

## Supplementary Material

Table S1

Table S2

Table S3

Table S4

## References

[bib97] Adams M.J., Blundell T.L., Dodson E.J., Dodson G.G., Vijayan M., Baker E.N., Harding M.M., Hodgkin D.C., Rimmer B., and Sheat S. 1969 Structure of Rhombohedral 2 Zinc Insulin Crystals. Nature. 224:491-495.0028-0836

[bib1] Afonine P.V., Poon B.K., Read R.J., Sobolev O.V., Terwilliger T.C., Urzhumtsev A., and Adams P.D. 2018 Real-space refinement in PHENIX for cryo-EM and crystallography. Acta Crystallogr. D Struct. Biol. 74:531–544. 10.1107/S2059798318006551 29872004 PMC6096492

[bib2] Alvarado D., Klein D.E., and Lemmon M.A. 2010 Structural basis for negative cooperativity in growth factor binding to an EGF receptor. Cell. 142:568–579. 10.1016/j.cell.2010.07.015 20723758 PMC2925043

[bib3] Baker E.N., Blundell T.L., Cutfield J.F., Cutfield S.M., Dodson E.J., Dodson G.G., Hodgkin D.M., Hubbard R.E., Isaacs N.W., Reynolds C.D., 1988 The structure of 2Zn pig insulin crystals at 1.5 A resolution. Philos. Trans. R. Soc. Lond. B Biol. Sci. 319:369–456. 10.1098/rstb.1988.0058 2905485

[bib4] Bartoschik T., Galinec S., Kleusch C., Walkiewicz K., Breitsprecher D., Weigert S., Muller Y.A., You C., Piehler J., Vercruysse T., 2018 Near-native, site-specific and purification-free protein labeling for quantitative protein interaction analysis by MicroScale Thermophoresis. Sci. Rep. 8:4977 10.1038/s41598-018-23154-3 29563556 PMC5862892

[bib5] Bass J., Kurose T., Pashmforoush M., and Steiner D.F. 1996 Fusion of insulin receptor ectodomains to immunoglobulin constant domains reproduces high-affinity insulin binding in vitro. J. Biol. Chem. 271:19367–19375. 10.1074/jbc.271.32.19367 8702623

[bib6] Belfiore A., and Malaguarnera R. 2011 Insulin receptor and cancer. Endocr. Relat. Cancer. 18:R125–R147. 10.1530/ERC-11-0074 21606157

[bib7] Bussi G., Donadio D., and Parrinello M. 2007 Canonical sampling through velocity rescaling. J. Chem. Phys. 126:014101 10.1063/1.2408420 17212484

[bib8] Chiacchia K.B. 1991 Quantitation of the class I disulfides of the insulin receptor. Biochem. Biophys. Res. Commun. 176:1178–1182. 10.1016/0006-291X(91)90409-Z 2039503

[bib9] Christiansen K., Tranum-Jensen J., Carlsen J., and Vinten J. 1991 A model for the quaternary structure of human placental insulin receptor deduced from electron microscopy. Proc. Natl. Acad. Sci. USA. 88:249–252. 10.1073/pnas.88.1.249 1986371 PMC50787

[bib10] Conway P., Tyka M.D., DiMaio F., Konerding D.E., and Baker D. 2014 Relaxation of backbone bond geometry improves protein energy landscape modeling. Protein Sci. 23:47–55. 10.1002/pro.2389 24265211 PMC3892298

[bib11] Coskun U., and Simons K. 2011 Cell membranes: the lipid perspective. Structure. 19:1543–1548. 10.1016/j.str.2011.10.010 22078554

[bib12] Croll T.I., Smith B.J., Margetts M.B., Whittaker J., Weiss M.A., Ward C.W., and Lawrence M.C. 2016 Higher-Resolution Structure of the Human Insulin Receptor Ectodomain: Multi-Modal Inclusion of the Insert Domain. Structure. 24:469–476. 10.1016/j.str.2015.12.014 26853939 PMC4860004

[bib13] Danne R., Poojari C., Martinez-Seara H., Rissanen S., Lolicato F., Rog T., and Vattulainen I. 2017 doGlycans-Tools for Preparing Carbohydrate Structures for Atomistic Simulations of Glycoproteins, Glycolipids, and Carbohydrate Polymers for GROMACS. J. Chem. Inf. Model. 57:2401–2406. 10.1021/acs.jcim.7b00237 28906114 PMC5662928

[bib14] Darden T., York D., and Pedersen L. 1993 Particle mesh Ewald: An N⋅log(N) method for Ewald sums in large systems. J. Chem. Phys. 98:10089–10092. 10.1063/1.464397

[bib15] De Meyts P. 1994 The structural basis of insulin and insulin-like growth factor-I receptor binding and negative co-operativity, and its relevance to mitogenic versus metabolic signalling. Diabetologia. 37:S135–S148. 10.1007/BF00400837 7821729

[bib16] De Meyts P. 2004 Insulin and its receptor: structure, function and evolution. BioEssays. 26:1351–1362. 10.1002/bies.20151 15551269

[bib17] De Meyts P. 2015 Insulin/receptor binding: the last piece of the puzzle? BioEssays. 37:389–397. 10.1002/bies.201400190 25630923

[bib18] De Meyts P., and Whittaker J. 2002 Structural biology of insulin and IGF1 receptors: implications for drug design. Nat. Rev. Drug Discov. 1:769–783. 10.1038/nrd917 12360255

[bib19] de Meyts P., Roth J., Neville D.M. Jr., Gavin J.R. III, and Lesniak M.A. 1973 Insulin interactions with its receptors: experimental evidence for negative cooperativity. Biochem. Biophys. Res. Commun. 55:154–161. 10.1016/S0006-291X(73)80072-5 4361269

[bib20] De Meyts P., Van Obberghen E., and Roth J. 1978 Mapping of the residues responsible for the negative cooperativity of the receptor-binding region of insulin. Nature. 273:504–509. 10.1038/273504a0 661960

[bib21] Delle Bovi R.J., and Miller W.T. 2017 Expression and purification of functional insulin and insulin-like growth factor 1 holoreceptors from mammalian cells. Anal. Biochem. 536:69–77. 10.1016/j.ab.2017.08.011 28830678 PMC5701837

[bib22] DeMeyts P., Bainco A.R., and Roth J. 1976 Site-site interactions among insulin receptors. Characterization of the negative cooperativity. J. Biol. Chem. 251:1877–1888. 5434

[bib23] Durocher Y., Perret S., and Kamen A. 2002 High-level and high-throughput recombinant protein production by transient transfection of suspension-growing human 293-EBNA1 cells. Nucleic Acids Res. 30:E9 10.1093/nar/30.2.e9 11788735 PMC99848

[bib24] Emsley P., Lohkamp B., Scott W.G., and Cowtan K. 2010 Features and development of Coot. Acta Crystallogr. D Biol. Crystallogr. 66:486–501. 10.1107/S0907444910007493 20383002 PMC2852313

[bib25] Endres N.F., Barros T., Cantor A.J., and Kuriyan J. 2014 Emerging concepts in the regulation of the EGF receptor and other receptor tyrosine kinases. Trends Biochem. Sci. 39:437–446. 10.1016/j.tibs.2014.08.001 25242369

[bib26] Essmann U., Perera L., Berkowitz M.L., Darden T., Lee H., and Pedersen L.G. 1995 A smooth particle mesh Ewald method. J. Chem. Phys. 103:8577–8593. 10.1063/1.470117

[bib27] Eswar N., Webb B., Marti-Renom M.A., Madhusudhan M.S., Eramian D., Shen M.Y., Pieper U., and Sali A. 2006 Comparative protein structure modeling using Modeller. Curr. Protoc. Bioinformatics. Chapter 5:Unit 5.6. 10.1002/0471250953.bi0506s15 PMC4186674 18428767

[bib28] Ferguson K.M., Berger M.B., Mendrola J.M., Cho H.S., Leahy D.J., and Lemmon M.A. 2003 EGF activates its receptor by removing interactions that autoinhibit ectodomain dimerization. Mol. Cell. 11:507–517. 10.1016/S1097-2765(03)00047-9 12620237

[bib29] Fernandes de Abreu D.A., Caballero A., Fardel P., Stroustrup N., Chen Z., Lee K., Keyes W.D., Nash Z.M., López-Moyado I.F., Vaggi F., 2014 An insulin-to-insulin regulatory network orchestrates phenotypic specificity in development and physiology. PLoS Genet. 10:e1004225 10.1371/journal.pgen.1004225 24675767 PMC3967928

[bib30] Fleishman S.J., Leaver-Fay A., Corn J.E., Strauch E.M., Khare S.D., Koga N., Ashworth J., Murphy P., Richter F., Lemmon G., 2011 RosettaScripts: a scripting language interface to the Rosetta macromolecular modeling suite. PLoS One. 6:e20161 10.1371/journal.pone.0020161 21731610 PMC3123292

[bib31] Freed D.M., Bessman N.J., Kiyatkin A., Salazar-Cavazos E., Byrne P.O., Moore J.O., Valley C.C., Ferguson K.M., Leahy D.J., Lidke D.S., and Lemmon M.A. 2017 EGFR Ligands Differentially Stabilize Receptor Dimers to Specify Signaling Kinetics. Cell. 171:683–695.e18. 10.1016/j.cell.2017.09.017 28988771 PMC5650921

[bib32] Garofalo R.S. 2002 Genetic analysis of insulin signaling in Drosophila. Trends Endocrinol. Metab. 13:156–162. 10.1016/S1043-2760(01)00548-3 11943559

[bib33] Gasteiger E., Gattiker A., Hoogland C., Ivanyi I., Appel R.D., and Bairoch A. 2003 ExPASy: The proteomics server for in-depth protein knowledge and analysis. Nucleic Acids Res. 31:3784–3788. 10.1093/nar/gkg563 12824418 PMC168970

[bib34] Goddard T.D., Huang C.C., Meng E.C., Pettersen E.F., Couch G.S., Morris J.H., and Ferrin T.E. 2018 UCSF ChimeraX: Meeting modern challenges in visualization and analysis. Protein Sci. 27:14–25. 10.1002/pro.3235 28710774 PMC5734306

[bib35] Gutmann T., Kim K.H., Grzybek M., Walz T., and Coskun Ü. 2018 Visualization of ligand-induced transmembrane signaling in the full-length human insulin receptor. J. Cell Biol. 217:1643–1649. 10.1083/jcb.201711047 29453311 PMC5940312

[bib36] Gutmann T., Schäfer I., Poojari C., Brankatschk B., Vattulainen I., Strauss M., and Coskun Ü. 2019 Cryo-EM structure of the complete and ligand-saturated insulin receptor ectodomain. bioRxiv. doi: (Preprint posted June 21, 2019). 10.1101/679233 PMC7039211 31727777

[bib37] Hess B., Bekker H., Berendsen H.J.C., and Fraaije J.G.E.M. 1997 LINCS: A linear constraint solver for molecular simulations. J. Comput. Chem. 18:1463–1472. 10.1002/(SICI)1096-987X(199709)18:12<1463::AID-JCC4>3.0.CO;2-H

[bib38] Hess B., Kutzner C., van der Spoel D., and Lindahl E. 2008 GROMACS 4: Algorithms for Highly Efficient, Load-Balanced, and Scalable Molecular Simulation. J. Chem. Theory Comput. 4:435–447. 10.1021/ct700301q 26620784

[bib39] Horwitz D.L., Starr J.I., Mako M.E., Blackard W.G., and Rubenstein A.H. 1975 Proinsulin, insulin, and C-peptide concentrations in human portal and peripheral blood. J. Clin. Invest. 55:1278–1283. 10.1172/JCI108047 1133173 PMC301883

[bib40] Hoyne P.A., Cosgrove L.J., McKern N.M., Bentley J.D., Ivancic N., Elleman T.C., and Ward C.W. 2000 High affinity insulin binding by soluble insulin receptor extracellular domain fused to a leucine zipper. FEBS Lett. 479:15–18. 10.1016/S0014-5793(00)01872-X 10940380

[bib41] Hua Q.X., Shoelson S.E., Kochoyan M., and Weiss M.A. 1991 Receptor binding redefined by a structural switch in a mutant human insulin. Nature. 354:238–241. 10.1038/354238a0 1961250

[bib42] Humphrey W., Dalke A., and Schulten K. 1996 VMD: visual molecular dynamics. J. Mol. Graph. 14:33–38. 10.1016/0263-7855(96)00018-5 8744570

[bib43] Jiráček J., and Žáková L. 2017 Structural Perspectives of Insulin Receptor Isoform-Selective Insulin Analogs. Front. Endocrinol. (Lausanne). 8:167 10.3389/fendo.2017.00167 28798723 PMC5529358

[bib44] Jorgensen W.L., Chandrasekhar J., Madura J.D., Impey R.W., and Klein M.L. 1983 Comparison of simple potential functions for simulating liquid water. J. Chem. Phys. 79:926–935. 10.1063/1.445869

[bib45] Kaminski G.A., Friesner R.A., Tirado-Rives J., and Jorgensen W.L. 2001 Evaluation and Reparametrization of the OPLS-AA Force Field for Proteins via Comparison with Accurate Quantum Chemical Calculations on Peptides†. J. Phys. Chem. B. 105:6474–6487. 10.1021/jp003919d

[bib46] Kasuga M., Zick Y., Blithe D.L., Crettaz M., and Kahn C.R. 1982 Insulin stimulates tyrosine phosphorylation of the insulin receptor in a cell-free system. Nature. 298:667–669. 10.1038/298667a0 6178977

[bib47] Kaszuba K., Grzybek M., Orłowski A., Danne R., Róg T., Simons K., Coskun Ü., and Vattulainen I. 2015 N-Glycosylation as determinant of epidermal growth factor receptor conformation in membranes. Proc. Natl. Acad. Sci. USA. 112:4334–4339. 10.1073/pnas.1503262112 25805821 PMC4394299

[bib48] Kavran J.M., McCabe J.M., Byrne P.O., Connacher M.K., Wang Z., Ramek A., Sarabipour S., Shan Y., Shaw D.E., Hristova K., 2014 How IGF-1 activates its receptor. eLife. 3:e03772 10.7554/eLife.03772 25255214 PMC4381924

[bib49] Kimanius D., Forsberg B.O., Scheres S.H., and Lindahl E. 2016 Accelerated cryo-EM structure determination with parallelisation using GPUs in RELION-2. eLife. 5:e18722 10.7554/eLife.18722 27845625 PMC5310839

[bib50] Kiselyov V.V., Versteyhe S., Gauguin L., and De Meyts P. 2009 Harmonic oscillator model of the insulin and IGF1 receptors’ allosteric binding and activation. Mol. Syst. Biol. 5:243 10.1038/msb.2008.78 19225456 PMC2657531

[bib51] Kleinridders A., Ferris H.A., Cai W., and Kahn C.R. 2014 Insulin action in brain regulates systemic metabolism and brain function. Diabetes. 63:2232–2243. 10.2337/db14-0568 24931034 PMC4066341

[bib52] Lata S., Reichel A., Brock R., Tampé R., and Piehler J. 2005 High-affinity adaptors for switchable recognition of histidine-tagged proteins. J. Am. Chem. Soc. 127:10205–10215. 10.1021/ja050690c 16028931

[bib53] Lax I., Mitra A.K., Ravera C., Hurwitz D.R., Rubinstein M., Ullrich A., Stroud R.M., and Schlessinger J. 1991 Epidermal growth factor (EGF) induces oligomerization of soluble, extracellular, ligand-binding domain of EGF receptor. A low resolution projection structure of the ligand-binding domain. J. Biol. Chem. 266:13828–13833. 1856216

[bib54] Leray V., Hubert P., Crémel G., and Staedel C. 1992 Detergents affect insulin binding, tyrosine kinase activity and oligomeric structure of partially purified insulin receptors. Arch. Biochem. Biophys. 294:22–29. 10.1016/0003-9861(92)90131-F 1312805

[bib55] Longo P.A., Kavran J.M., Kim M.S., and Leahy D.J. 2013 Transient mammalian cell transfection with polyethylenimine (PEI). Methods Enzymol. 529:227–240. 10.1016/B978-0-12-418687-3.00018-5 24011049 PMC4012321

[bib56] Luo R.Z., Beniac D.R., Fernandes A., Yip C.C., and Ottensmeyer F.P. 1999 Quaternary structure of the insulin-insulin receptor complex. Science. 285:1077–1080. 10.1126/science.285.5430.1077 10446056

[bib57] Mastronarde D.N. 2005 Automated electron microscope tomography using robust prediction of specimen movements. J. Struct. Biol. 152:36–51. 10.1016/j.jsb.2005.07.007 16182563

[bib58] McGreevy R., Teo I., Singharoy A., and Schulten K. 2016 Advances in the molecular dynamics flexible fitting method for cryo-EM modeling. Methods. 100:50–60. 10.1016/j.ymeth.2016.01.009 26804562 PMC4848153

[bib59] McKern N.M., Lawrence M.C., Streltsov V.A., Lou M.Z., Adams T.E., Lovrecz G.O., Elleman T.C., Richards K.M., Bentley J.D., Pilling P.A., 2006 Structure of the insulin receptor ectodomain reveals a folded-over conformation. Nature. 443:218–221. 10.1038/nature05106 16957736

[bib60] Menting J.G., Whittaker J., Margetts M.B., Whittaker L.J., Kong G.K., Smith B.J., Watson C.J., Záková L., Kletvíková E., Jiráček J., 2013 How insulin engages its primary binding site on the insulin receptor. Nature. 493:241–245. 10.1038/nature11781 23302862 PMC3793637

[bib61] Menting J.G., Yang Y., Chan S.J., Phillips N.B., Smith B.J., Whittaker J., Wickramasinghe N.P., Whittaker L.J., Pandyarajan V., Wan Z.L., 2014 Protective hinge in insulin opens to enable its receptor engagement. Proc. Natl. Acad. Sci. USA. 111:E3395–E3404. 10.1073/pnas.1412897111 25092300 PMC4143003

[bib62] Muggeo M., Van Obberghen E., Kahn C.R., Roth J., Ginsberg B.H., De Meyts B.H., Emdin S.O., and Falkmer S. 1979 The insulin receptor and insulin of the Atlantic hagfish. Extraordinary conservation of binding specificity and negative cooperativity in the most primitive vertebrate. Diabetes. 28:175–181. 10.2337/diab.28.3.175 446902

[bib63] Parrinello M., and Rahman A. 1980 Crystal Structure and Pair Potentials: A Molecular-Dynamics Study. Phys. Rev. Lett. 45:1196–1199. 10.1103/PhysRevLett.45.1196

[bib64] Parrinello M., and Rahman A. 1981 Polymorphic transitions in single crystals: A new molecular dynamics method. J. Appl. Phys. 52:7182–7190. 10.1063/1.328693

[bib65] Parrinello M., and Rahman A. 1982 Strain fluctuations and elastic constants. J. Chem. Phys. 76:2662–2666. 10.1063/1.443248

[bib66] Pettersen E.F., Goddard T.D., Huang C.C., Couch G.S., Greenblatt D.M., Meng E.C., and Ferrin T.E. 2004 UCSF Chimera--a visualization system for exploratory research and analysis. J. Comput. Chem. 25:1605–1612. 10.1002/jcc.20084 15264254

[bib67] Rentería M.E., Gandhi N.S., Vinuesa P., Helmerhorst E., and Mancera R.L. 2008 A comparative structural bioinformatics analysis of the insulin receptor family ectodomain based on phylogenetic information. PLoS One. 3:e3667 10.1371/journal.pone.0003667 18989367 PMC2577065

[bib68] Rigaut G., Shevchenko A., Rutz B., Wilm M., Mann M., and Séraphin B. 1999 A generic protein purification method for protein complex characterization and proteome exploration. Nat. Biotechnol. 17:1030–1032. 10.1038/13732 10504710

[bib69] Rosenthal P.B., and Henderson R. 2003 Optimal determination of particle orientation, absolute hand, and contrast loss in single-particle electron cryomicroscopy. J. Mol. Biol. 333:721–745. 10.1016/j.jmb.2003.07.013 14568533

[bib70] Saltiel A.R., and Kahn C.R. 2001 Insulin signalling and the regulation of glucose and lipid metabolism. Nature. 414:799–806. 10.1038/414799a 11742412

[bib71] Scapin G., Dandey V.P., Zhang Z., Prosise W., Hruza A., Kelly T., Mayhood T., Strickland C., Potter C.S., and Carragher B. 2018 Structure of the insulin receptor-insulin complex by single-particle cryo-EM analysis. Nature. 556:122–125. 10.1038/nature26153 29512653 PMC5886813

[bib72] Schäffer L. 1994 A model for insulin binding to the insulin receptor. Eur. J. Biochem. 221:1127–1132. 10.1111/j.1432-1033.1994.tb18833.x 8181471

[bib73] Seidel S.A., Dijkman P.M., Lea W.A., van den Bogaart G., Jerabek-Willemsen M., Lazic A., Joseph J.S., Srinivasan P., Baaske P., Simeonov A., 2013 Microscale thermophoresis quantifies biomolecular interactions under previously challenging conditions. Methods. 59:301–315. 10.1016/j.ymeth.2012.12.005 23270813 PMC3644557

[bib74] Sparrow L.G., McKern N.M., Gorman J.J., Strike P.M., Robinson C.P., Bentley J.D., and Ward C.W. 1997 The disulfide bonds in the C-terminal domains of the human insulin receptor ectodomain. J. Biol. Chem. 272:29460–29467. 10.1074/jbc.272.47.29460 9368005

[bib75] Sparrow L.G., Gorman J.J., Strike P.M., Robinson C.P., McKern N.M., Epa V.C., and Ward C.W. 2007 The location and characterisation of the O-linked glycans of the human insulin receptor. Proteins. 66:261–265. 10.1002/prot.21261 17078079

[bib76] Sparrow L.G., Lawrence M.C., Gorman J.J., Strike P.M., Robinson C.P., McKern N.M., and Ward C.W. 2008 N-linked glycans of the human insulin receptor and their distribution over the crystal structure. Proteins. 71:426–439. 10.1002/prot.21768 17957771

[bib77] Subramanian K., Fee C.J., Fredericks R., Stubbs R.S., and Hayes M.T. 2013 Insulin receptor-insulin interaction kinetics using multiplex surface plasmon resonance. J. Mol. Recognit. 26:643–652. 10.1002/jmr.2307 24277609

[bib78] Tatulian S.A. 2015 Structural Dynamics of Insulin Receptor and Transmembrane Signaling. Biochemistry. 54:5523–5532. 10.1021/acs.biochem.5b00805 26322622

[bib79] Trabuco L.G., Villa E., Schreiner E., Harrison C.B., and Schulten K. 2009 Molecular dynamics flexible fitting: a practical guide to combine cryo-electron microscopy and X-ray crystallography. Methods. 49:174–180. 10.1016/j.ymeth.2009.04.005 19398010 PMC2753685

[bib80] Tranum-Jensen J., Christiansen K., Carlsen J., Brenzel G., and Vinten J. 1994 Membrane topology of insulin receptors reconstituted into lipid vesicles. J. Membr. Biol. 140:215–223. 10.1007/BF00233710 7932656

[bib81] Uchikawa E., Choi E., Shang G., Yu H., and Bai X.C. 2019 Activation mechanism of the insulin receptor revealed by cryo-EM structure of the fully liganded receptor-ligand complex. eLife. 8:e48630 10.7554/eLife.48630 31436533 PMC6721835

[bib82] Wang Z., and Schröder G.F. 2012 Real-space refinement with DireX: from global fitting to side-chain improvements. Biopolymers. 97:687–697. 10.1002/bip.22046 22696405

[bib83] Weis F., Menting J.G., Margetts M.B., Chan S.J., Xu Y., Tennagels N., Wohlfart P., Langer T., Müller C.W., Dreyer M.K., and Lawrence M.C. 2018 The signalling conformation of the insulin receptor ectodomain. Nat. Commun. 9:4420 10.1038/s41467-018-06826-6 30356040 PMC6200814

[bib84] Weiss M.A., and Lawrence M.C. 2018 A thing of beauty: Structure and function of insulin’s “aromatic triplet”. Diabetes Obes. Metab. 20:51–63. 10.1111/dom.13402 30230175 PMC6159917

[bib85] Whittaker J., Garcia P., Yu G.Q., and Mynarcik D.C. 1994 Transmembrane domain interactions are necessary for negative cooperativity of the insulin receptor. Mol. Endocrinol. 8:1521–1527. 7877620 10.1210/mend.8.11.7877620

[bib86] Whittaker L., Hao C., Fu W., and Whittaker J. 2008 High-affinity insulin binding: insulin interacts with two receptor ligand binding sites. Biochemistry. 47:12900–12909. 10.1021/bi801693h 18991400 PMC2819479

[bib87] Woldin C.N., Hing F.S., Lee J., Pilch P.F., and Shipley G.G. 1999 Structural studies of the detergent-solubilized and vesicle-reconstituted insulin receptor. J. Biol. Chem. 274:34981–34992. 10.1074/jbc.274.49.34981 10574975

[bib88] Xu B., Huang K., Chu Y.C., Hu S.Q., Nakagawa S., Wang S., Wang R.Y., Whittaker J., Katsoyannis P.G., and Weiss M.A. 2009 Decoding the cryptic active conformation of a protein by synthetic photoscanning: insulin inserts a detachable arm between receptor domains. J. Biol. Chem. 284:14597–14608. 10.1074/jbc.M900087200 19321435 PMC2682907

[bib89] Xu Y., Kong G.K., Menting J.G., Margetts M.B., Delaine C.A., Jenkin L.M., Kiselyov V.V., De Meyts P., Forbes B.E., and Lawrence M.C. 2018 How ligand binds to the type 1 insulin-like growth factor receptor. Nat. Commun. 9:821 10.1038/s41467-018-03219-7 29483580 PMC5826941

[bib90] Ye L., Maji S., Sanghera N., Gopalasingam P., Gorbunov E., Tarasov S., Epstein O., and Klein-Seetharaman J. 2017 Structure and dynamics of the insulin receptor: implications for receptor activation and drug discovery. Drug Discov. Today. 22:1092–1102. 10.1016/j.drudis.2017.04.011 28476537

[bib91] Zhang K. 2016 Gctf: Real-time CTF determination and correction. J. Struct. Biol. 193:1–12. 10.1016/j.jsb.2015.11.003 26592709 PMC4711343

[bib92] Zheng S.Q., Palovcak E., Armache J.P., Verba K.A., Cheng Y., and Agard D.A. 2017 MotionCor2: anisotropic correction of beam-induced motion for improved cryo-electron microscopy. Nat. Methods. 14:331–332. 10.1038/nmeth.4193 28250466 PMC5494038

[bib93] Zivanov J., Nakane T., Forsberg B.O., Kimanius D., Hagen W.J., Lindahl E., and Scheres S.H. 2018 New tools for automated high-resolution cryo-EM structure determination in RELION-3. eLife. 7:e42166 10.7554/eLife.42166 30412051 PMC6250425

